# Real-World Outcomes of Switching to Aflibercept 8 mg in Previously Treated Neovascular Age-Related Macular Degeneration: A Systematic Review and Meta-Analysis

**DOI:** 10.3390/jcm15124599

**Published:** 2026-06-13

**Authors:** Abdullah Bousamri, Mohammad Kana’an, Faisal Alharbi, Noor Alqudah

**Affiliations:** 1 Faculty of Medicine, Jordan University of Science and Technology, Irbid 22110, Jordan; 2 Department of Ophthalmology, Farwaniya Hospital, Farwaniya Governorate, Farwaniya Area, Farwaniya 81004, Kuwait; 3 Division of Ophthalmology, Department of Special Surgery, Faculty of Medicine, Jordan University of Science and Technology, Irbid 22110, Jordan; nmalqudah5@just.edu.jo

**Keywords:** aflibercept 8 mg, neovascular age-related macular degeneration, anti-VEGF, switching, best-corrected visual acuity, central subfield thickness, treatment interval

## Abstract

**Background**: Neovascular age-related macular degeneration (nAMD) remains a leading cause of irreversible central vision loss. Although anti-vascular endothelial growth factor (anti-VEGF) therapy has transformed management, pivotal trials enrolled exclusively treatment-naïve patients, leaving clinicians without pooled evidence to guide switching decisions in previously treated eyes. This systematic review and meta-analysis assessed real-world visual, anatomical, durability, and safety outcomes following switching to aflibercept 8 mg in previously treated nAMD. **Methods**: Following PRISMA 2020 guidelines, we searched PubMed, Embase, Web of Science, CENTRAL, Scopus, and Google Scholar through April 2026. Studies reporting switching to aflibercept 8 mg with change in best-corrected visual acuity (BCVA), central subfield thickness (CST), or treatment interval were included. Continuous outcomes were pooled using random-effects models with Hartung–Knapp–Sidik–Jonkman adjustment; proportions were estimated using generalized linear mixed models. Methodological quality was evaluated using the JBI Critical Appraisal Checklist for Case Series. Certainty of evidence was assessed using GRADE. The protocol was registered with PROSPERO (CRD420261371334). **Results**: Twenty-one studies met inclusion criteria. BCVA remained stable (WMD: −0.017 logMAR; 95% CI: −0.027 to −0.007; +0.83 ETDRS letters; I^2^ = 0%). CST decreased significantly (WMD: −21.5 µm; 95% CI: −29.3 to −13.7; I^2^ = 56.0%), and treatment intervals extended by +1.79 weeks (95% CI: +1.32 to +2.27; I^2^ = 74.3%). Intraretinal and subretinal fluid each resolved in 37.5% of eyes. Intraocular inflammation was rare across 9959 treated eyes, though this pool was not restricted to switched eyes, with no confirmed retinal vasculitis. Sensitivity analyses confirmed robustness across all co-primary estimates. GRADE certainty was low for BCVA and very low for CST and treatment interval. **Conclusions**: Low-certainty evidence suggests that switching to aflibercept 8 mg preserves visual acuity, while very-low-certainty evidence suggests reductions in central subfield thickness and modest extension of treatment intervals. Intraocular inflammation was rare, though safety denominators included non-switch eyes. These findings provide preliminary pooled estimates to inform switch decisions in previously treated eyes.

## 1. Introduction

Neovascular age-related macular degeneration (nAMD) remains the leading cause of irreversible central vision loss among the elderly population. It is estimated that 196 million people were affected globally in 2020, and it is projected to reach up to 288 million by 2040 [1,2]. The disease is characterized by abnormal growth of choroidal neovascular membranes beneath the retina, driven by overexpression of vascular endothelial growth factor (VEGF). This activity leads to exudation of fluid and blood into the intraretinal and subretinal spaces, resulting in subsequent photoreceptor damage [2]. Anti-vascular endothelial growth factor (anti-VEGF) therapy has transformed disease management with drugs such as ranibizumab, bevacizumab, aflibercept 2 mg, brolucizumab, and faricimab. These agents demonstrate the capacity to stabilize or improve visual acuity by suppressing pathological choroidal neovascularization [3,4,5,6,7]. However, effective nAMD management imposes a substantial treatment burden. Most eyes require indefinite intravitreal injections at four to eight-week intervals, although faricimab has demonstrated dosing intervals of up to 16 weeks in clinical trials [7]. Even with optimized treat-and-extend regimens, many patients do not achieve meaningful interval extension beyond this range [8]. Despite the ongoing therapy, it is very common for intraretinal and subretinal fluid to persist or recur [9]. Many factors contribute to inferior real-world visual outcomes compared with those observed in clinical trials [10], including the cumulative demands of frequent clinic visits and injections, which lead to patients’ nonadherence and treatment discontinuation.

The development of higher-dose anti-VEGF therapy was intended to ease the burden of these injections. Aflibercept 8 mg was designed to achieve a fourfold higher molar concentration than the standard dose of 2 mg [11] and received regulatory approval from the United States Food and Drug Administration in August 2023 and from the European Medicines Agency in January 2024. After providing promising results reported in trials such as the Phase 2 CANDELA trial, which demonstrated superior anatomical outcomes, including greater fluid resolution, for aflibercept 8 mg compared with 2 mg in treatment-naïve nAMD [11], the Phase 3 PULSAR trial also established the noninferiority of aflibercept 8 mg administered at 12- or 16-week intervals compared with aflibercept 2 mg given every 8 weeks in treatment-naïve eyes, with most patients maintaining extended dosing intervals at 1 year [12]. Yet the switch population differs fundamentally from that in these trials. It includes challenging cases in clinical practice, such as eyes with years of chronic disease, multiple prior injections, and residual fluid persisting through multiple lines of therapy. For this population, the clinical decision to switch cannot rely solely on trial data, and a systematic synthesis of real-world switching outcomes is needed to inform evidence-based practice.

After regulatory approval, the number of real-world studies assessing outcomes of switching from previously treated nAMD eyes to aflibercept 8 mg increased [13,14,15,16,17,18,19,20,21,22,23,24,25,26,27,28,29,30,31,32,33]. These studies remain fragmented, use different methodologies, and lack a systematic synthesis of this evidence.

This systematic review and meta-analysis provide the first pooled evidence from real-world outcomes of switching from prior anti-VEGF therapy to aflibercept 8 mg in previously treated nAMD. The objectives are mainly three co-primary outcomes: (1) change in best corrected visual acuity (BCVA), (2) change in central subfield thickness (CST), and (3) change in treatment interval. Additionally, secondary outcomes including intraretinal fluid (IRF) resolution; subretinal fluid (SRF) resolution; pigment epithelial detachment (PED) height change; proportion of eyes achieving treatment intervals from week 8, 12, and 16 or longer; intraocular inflammation (IOI) incidence; and all-cause discontinuation were assessed to provide clear evidence for clinicians to inform switch decisions.

## 2. Materials and Methods

### 2.1. Protocol and Registration

This review adhered to the Preferred Reporting Items for Systematic Reviews and Meta-Analyses PRISMA 2020 and the Cochrane Handbook for Systematic Reviews of Interventions [34,35]. The completed PRISMA 2020 checklist is attached in the Supplementary File. The protocol was registered with PROSPERO at CRD420261371334, with amendments documented in the registry record.

### 2.2. Search Strategy

An extensive literature search was conducted from inception through 24 April 2026, using the following databases: PubMed/Medline, Web of Science, Cochrane Central Register of Controlled Trials (CENTRAL), Scopus, Embase, and the first 200 results from Google Scholar, sorted by relevance, to capture gray literature. A registry such as Clinicaltrials.gov was also covered. No language or date restrictions were applied. The search strategy relied on the combination of Medical Subject Headings (MeSH) terms and free-text keywords related to the two core concepts: (1) the Intervention (“Aflibercept 8 mg,” “high dose aflibercept,” “Eylea HD”); (2) the population (“Macular Degeneration,” “Wet Macular Degeneration,” “neovascular age-related macular degeneration”). The same concepts were applied to the remaining databases, with the syntax adjusted and entries corrected for each. Detailed search strings used are provided in the Supplementary File.

### 2.3. Inclusion and Exclusion Criteria

Two reviewers independently screened the titles and abstracts using set eligibility criteria. Studies were included if they involved: (1) adult patients with neovascular age-related macular degeneration who had previously received any anti-VEGF treatment and were switched to intravitreal aflibercept 8 mg; (2) reported quantitative data on at least one of the main outcomes, such as change in best-corrected visual acuity, central subfield thickness, or treatment interval, along with a measure of dispersion (standard deviation, confidence interval, interquartile range, or standard error) to allow measurement of a weighted mean difference; and (3) were either prospective or retrospective observational studies with at least 10 eyes in the switch group.

Studies were excluded if they met any of the following: (1) they were case reports, reviews, meta-analyses, editorials, conference abstracts, or letters without original research data; (2) they did not provide extractable outcome data or measures of dispersion for any co-primary endpoint; (3) they focused only on treatment-naïve patients or included both treatment-naïve and previously treated groups where the switch cohort could not be analyzed separately; (4) they enrolled fewer than 10 eyes in the switch cohort. We included one study published as a letter to the editor [14] because it contained original research with complete methods, results, and ethical approval, and met all other eligibility criteria.

Disagreements between reviewers during screening and eligibility assessment were resolved through discussion and consultation with a third reviewer when necessary. The study selection process is detailed in the Results section.

### 2.4. Data Extraction

Two reviewers independently extracted data from the included studies using a prespecified standardized Excel spreadsheet. After piloting in three studies, full-scale implementation was carried out in the remaining studies. Any disagreements were resolved through thorough discussion and input from a third reviewer. For each included study, the following data were collected: first author, publication year, country of origin, study design, sample size, demographic characteristics (mean age, sex distribution), baseline clinical parameters (best-corrected visual acuity, central subfield thickness, treatment interval, disease duration, number of prior anti-VEGF injections, prior anti-VEGF agent), intervention details (loading phase protocol, treat-and-extend regimen, dosing schedule), and all prespecified primary and secondary outcome measures with accompanying measures of dispersion. The eye was the unit of analysis throughout, consistent with the reporting conventions of the included studies. For studies reporting outcomes in ETDRS letters, conversion to *logMAR* was performed using the Gregori formula: $logMAR=\;\frac{85-ETDRS}{50}$ [36]. For studies reporting medians with interquartile ranges, means and standard deviations were estimated using the methods of Wan et al. [37] and Luo et al. [38]. If the standard deviation of within-eye change was unavailable, an estimate from the baseline and final standard deviations was made, assuming a pre-post correlation of r = 0.5; sensitivity analyses were performed at r = 0.3 and r = 0.7 [35]. For PED height, an additional sensitivity at r = 0.8 was performed given the high temporal stability of PED measurements. If a study had both previously treated and treatment-naïve eyes, subgroup-specific data for the switch cohort were extracted if possible. For studies with results at different follow-up time points, the last available time point was used as the primary analysis input. If some data were missing or unclear, an attempt was made to contact the study authors for clarification. If they did not respond after two emails over four weeks, we included the study in our analysis using the best available data and noted this limitation.

### 2.5. Quality Assessment

Two reviewers assessed the methodological quality of the included studies independently using the JBI Critical Appraisal Checklist for Case Series [39]. This tool evaluates 10 items: inclusion criteria, case identification and measurement, participant selection and completeness, demographic and clinical reporting, and statistical analysis. Each item was scored as 1 (“yes”) or 0 (“no,” “unclear,” or “not applicable”), yielding a total percentage out of 10 applicable items. Scores ≥80% were considered low risk of bias, 50–79% moderate risk, and <50% high risk. No studies were excluded on the basis of methodological quality; rather, these ratings were used to guide sensitivity analyses and contextualize the interpretation of findings. Disagreements between reviewers were resolved by consensus.

### 2.6. Statistical Analysis

Meta-analyses were conducted using R (version 4.5.2; R Foundation for Statistical Computing, Vienna, Austria) with the metafor package (version 4.8-0) [40]. Two adopted models were used for the two types of outcomes: (1) Continuous outcomes such as BCVA change, CST change, treatment interval change, PED height change, and pooled weighted mean differences with 95% confidence intervals using random-effects models with the DerSimonian–Laird (DL) estimator [41] and Hartung–Knapp–Sidik–Jonkman (HKSJ) adjustment [42]; DL was selected because restricted maximum likelihood failed to converge for several outcomes. (2) Proportion outcomes such as IRF and SRF resolution; interval achievement at 8, 12, and 16 weeks; IOI incidence; and discontinuation pooled proportions were estimated using generalized linear mixed models with a logit link function as the primary method, with Freeman–Tukey double arcsine transformation [43] as a prespecified sensitivity analysis. For safety outcomes, the denominator included all eyes receiving aflibercept 8 mg in each study regardless of treatment history or indication, as several studies reported inflammation events at the cohort level without separating switched eyes.

Between-study heterogeneity was quantified using Cochran’s Q statistic (significance at *p* < 0.10) and the I^2^ statistic. Prespecified subgroup analyses for co-primary outcomes examined geographic region, follow-up duration, reason for switch, loading phase, regulatory label constraint, and prior aflibercept 2 mg majority. Residual I^2^ was reported for each moderator. The nAMD subtype subgroup was prespecified but could not be performed due to insufficient subtype-specific reporting.

Sensitivity analyses included exclusion of studies with fewer than 20 eyes, follow-up under 3 months, single-injection assessments, label-constraint jurisdictions, and restriction to native ETDRS reporters for BCVA. Correlation sensitivity was tested using imputed standard deviations at r = 0.3 and r = 0.7, alongside the primary r = 0.5. Leave-one-out analyses assessed the influence of individual studies on pooled estimates. Publication bias was evaluated using funnel plots with Egger’s test for continuous outcomes or Peters’ test for proportions, applied only where 10 or more studies contributed.

### 2.7. Certainty of Evidence

The certainty of evidence for each co-primary outcome was assessed using the Grading of Recommendations, Assessment, Development and Evaluation (GRADE) framework [35]. Evidence from observational studies started at low certainty and was further rated down for risk of bias, inconsistency, indirectness, imprecision, or publication bias, and rated up for large effect size, dose–response gradient, or plausible residual confounding that would reduce the observed effect. Two reviewers independently evaluated each domain, and disagreements were resolved through discussion. Certainty ratings were classified as high, moderate, low, or very low. The complete GRADE assessment is presented in Supplementary Table S1.

## 3. Results

### 3.1. Study Selection

The comprehensive search across six databases and one registry identified 732 records: PubMed/MEDLINE (*n* = 97), Web of Science (*n* = 119), CENTRAL (*n* = 42), Scopus (*n* = 93), Embase (*n* = 169), Google Scholar (*n* = 200), and Clinicaltrial.gov (*n* = 12). Using EndNote X9 (Clarivate, Philadelphia, PA, USA), deduplication yielded 411 unique results. Of these, 51 were eligible for full-text screening. One was inaccessible, and 50 were fully read for eligibility by two reviewers.

**Figure 1:**
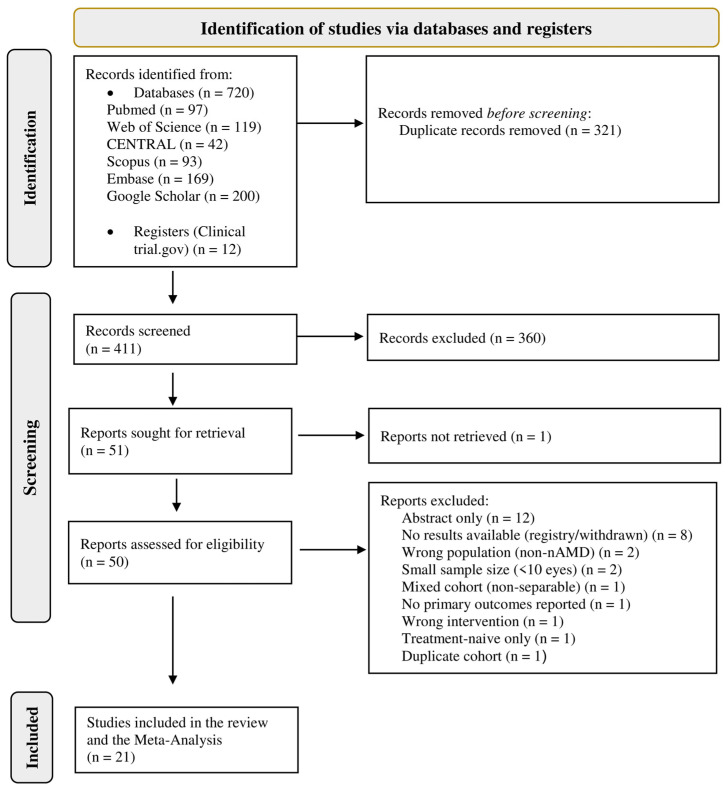
PRISMA 2020 flow diagram of the included studies.

Twenty-one studies matched the prespecified eligibility criteria; 29 were excluded due to these reasons: abstract only without full-text data (*n* = 12), trials that had not finished yet or were withdrawn (*n* = 8), wrong population not involving nAMD (*n* = 2), small sample size with fewer than 10 eyes (*n* = 2), mixed cohort with non-separable switch data (*n* = 1), no primary outcomes reported (*n* = 1), wrong intervention (*n* = 1), treatment-naïve only population (*n* = 1), and duplicate cohort (*n* = 1). Full reasons for exclusion are provided in the Supplementary Table S2. A flow diagram of the process is shown in Figure 1.

### 3.2. Study and Population Characteristics

Twenty-one studies met the inclusion criteria [13,14,15,16,17,18,19,20,21,22,23,24,25,26,27,28,29,30,31,32,33], encompassing 6448 eyes from 10 countries across 4 continents. One additional global multicenter cohort included 18 countries [15]. All studies were retrospective, except for one prospective observational study [15]. Sample sizes ranged from 15 [28] to 246 eyes [27] across the co-primary efficacy analyses, while the largest cohort (4823 eyes [17]) contributed only to the safety analysis. The studies came from the following: eight in Europe [13,20,21,22,24,27,31,32], five in Japan [23,25,26,28,29], four in the United States [16,17,30,33], and one each in Australia [14], Singapore [18], Taiwan [19], and a global cohort [15]. Participants’ average ages ranged from 69 to 82 years. Prior anti-VEGF treatment varied, with median or mean injection counts ranging from 9 [13] to 60 [32]. Most patients had previously received aflibercept 2 mg, followed by faricimab, ranibizumab, bevacizumab, and brolucizumab. Baseline CST ranged from 230.5 µm [24] to 371.6 µm [21]. Most studies used a treat-and-extend regimen without a loading phase, but two studies included a loading phase after switching [21,29]. Follow-up periods ranged from 1.0 months [28] to 12.0 months [27,29]. Full study and baseline details are in Table 1 and Table 2.

### 3.3. Results of Quality Assessment

Of 21 studies, 20 (95.2%) had low risk of bias, and one (4.8%) had moderate risk [33]. All studies met criteria for inclusion: clarity, condition measurement, case identification, demographic reporting, clinical information, outcome reporting, and site demographics. The most frequent limitations were absent consecutive enrollment statements in 11 studies (52.4%), incomplete participant inclusion documentation in 14 (66.7%), and inadequate statistical analysis in 2 (9.5%) due to missing formal testing or dispersion measures [14,33]. Consistent strengths included clear nAMD diagnostic criteria, validated outcome instruments, and adequate clinical reporting. Primary limitations inherent to the case series design were the absence of comparator groups, variable follow-up durations, and incomplete screening documentation. Individual study ratings are presented in Figure S1.

### 3.4. Best Corrected Visual Acuity

**Figure 2:**
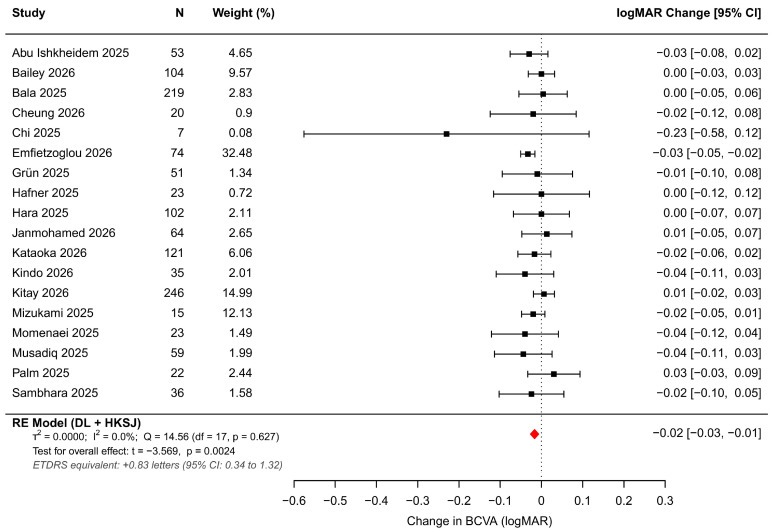
Forest plot of pooled weighted mean difference in BCVA (logMAR) after switching to aflibercept 8 mg (k = 18; N = 1274 eyes) [13,15,16,18,19,20,21,22,23,24,25,26,27,28,30,31,32,33].

Eighteen studies [13,15,16,18,19,20,21,22,23,24,25,26,27,28,30,31,32,33], covering 1274 eyes, had sufficient data to combine results for BCVA change. The combined weighted mean difference was −0.017 logMAR (95% CI: −0.027 to −0.007; *p* = 0.002), which equals a gain of 0.83 ETDRS letters (95% CI: +0.34 to +1.32) (Figure 2), indicating stable visual acuity after switching to aflibercept 8 mg. There was no heterogeneity (I^2^ = 0.0%; Q = 14.56, df = 17, *p* = 0.628). Per-outcome exclusion reasons for all co-primary outcomes are detailed in the Supplementary File.

### 3.5. Central Subfield Thickness

**Figure 3:**
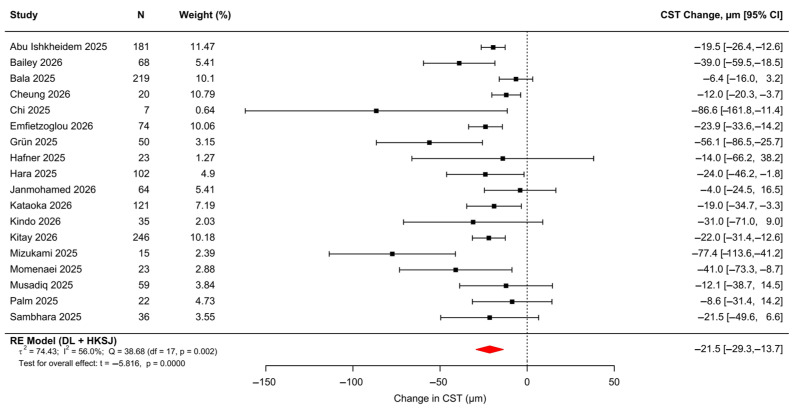
Forest plot of pooled change in CST (µm) after switching to aflibercept 8 mg (k = 18; 1365 eyes) [13,15,16,18,19,20,21,22,23,24,25,26,27,28,30,31,32,33].

Eighteen studies [13,15,16,18,19,20,21,22,23,24,25,26,27,28,30,31,32,33], covering 1365 eyes, were pooled. WMD was −21.5 µm (95% CI: −29.3 to −13.7; *p* < 0.001) (Figure 3), representing a statistically significant reduction in central subfield thickness after switching. The heterogeneity was moderate at (I^2^ = 56.0%; Q = 38.68, df = 17, *p* = 0.002).

### 3.6. Treatment Interval

**Figure 4:**
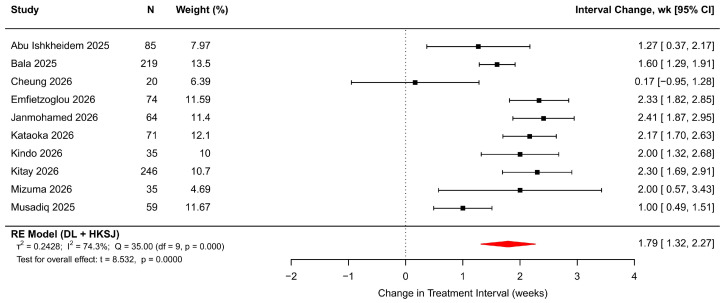
Forest plot of pooled change in treatment interval (weeks) after switching to aflibercept 8 mg (k = 10; 908 eyes) [13,16,18,20,24,25,26,27,29,31].

Ten studies [13,16,18,20,24,25,26,27,29,31], including 908 eyes, provided sufficient data to pool treatment interval changes. The pooled WMD was +1.79 weeks (95% CI: +1.32 to +2.27; *p* < 0.001) (Figure 4), indicating a significant extension of treatment intervals following switching. Substantial heterogeneity was present (I^2^ = 74.3%; Q = 35.00, df = 9, *p* < 0.001).

### 3.7. Secondary and Safety Outcomes

**Figure 5:**
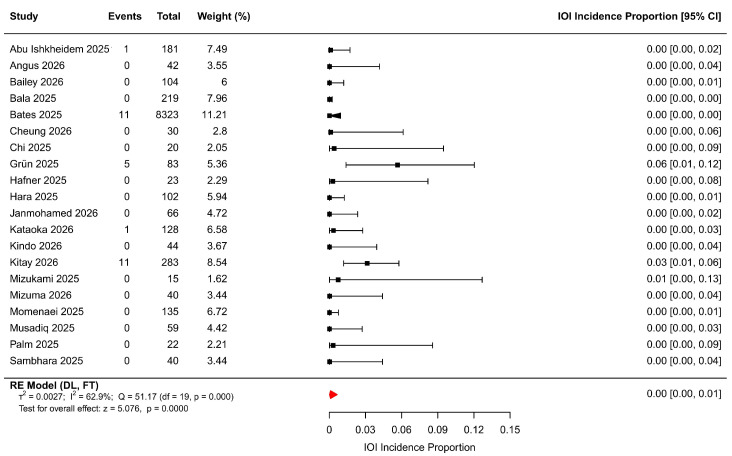
Forest plot of pooled intraocular inflammation incidence after switching to aflibercept 8 mg (k = 20; 9959 eyes). The pooled estimate displayed reflects the Freeman–Tukey transformation; the primary GLMM-logit estimate is reported in the text [13,14,15,16,17,18,19,21,22,23,24,25,26,27,28,29,30,31,32,33].

In 20 studies that provided safety data for 9959 eyes, the pooled incidence of IOI was 0.2% (95% CI: 0.0 to 1.0). This denominator is not restricted to switched eyes, as several studies reported inflammation events at the whole-cohort level. Fifteen studies reported no events, and no confirmed cases of retinal vasculitis were reported (Figure 5). The pooled all-cause treatment discontinuation rate was 19.9% (95% CI: 13.3 to 28.7; k = 9; I^2^ = 86.0%).

For secondary anatomical outcomes, the pooled proportion of IRF resolution was 37.5% (95% CI: 27.7 to 48.5; k = 12), and SRF resolution was also 37.5% (95% CI: 29.7 to 46.1; k = 11) based on GLMM-logit analysis. The pooled weighted mean difference in PED height was −22.9 µm (95% CI: −31.2 to −14.6; *p* < 0.001; k = 9; I^2^ = 35.4%). Regarding durability, 69.8% of eyes reached intervals of at least 8 weeks (k = 8), 13.7% reached 12 weeks or more (k = 6), and 9.6% reached 16 weeks or more (k = 4). Freeman–Tukey sensitivity analyses produced results similar to the GLMM estimates for all proportion outcomes (Supplementary Table S3). Supplementary Figures S2–S5 show forest plots for all secondary and safety outcomes.

### 3.8. Sensitivity and Subgroup Analyses

The pooled estimates for all three main outcomes stayed consistent across the planned sensitivity analyses. These included removing small studies, short follow-up periods, single-injection assessments, and studies from label-constraint jurisdictions (Supplementary Table S4). The BCVA estimate was no longer statistically significant when only studies using ETDRS letters were included (WMD = +0.002; *p* = 0.80; k = 7), which suggests that converting units may reduce measurement accuracy. Sensitivity analyses using correlation values of r = 0.3 and r = 0.7 did not change any of the main estimates. Leave-one-out analyses showed that no single study had a major impact on the pooled results for BCVA, CST, or treatment interval (Figure S6). In subgroup analyses, none of the six planned moderators greatly reduced the remaining heterogeneity for CST (residual I^2^ ranged from 54.6 to 58.4%). For the treatment interval, the reason for switching partly explained the heterogeneity (residual I^2^ = 54.5%, down from 74.3%), with refractory eyes showing a greater increase in interval (+2.23 weeks) compared to those with persistent disease (+1.16 weeks). The length of follow-up also played a role (residual I^2^ = 59.1%). Full subgroup results are available in Supplementary Tables S5–S8.

### 3.9. Publication Bias

Funnel plots and regression tests revealed no significant asymmetry for BCVA (Egger *p* = 0.771) or treatment interval (Egger *p* = 0.783). Borderline asymmetry was observed for CST (Egger *p* = 0.048); further, Duval and Tweedie trim-and-fill analysis imputed 4 missing studies; the adjusted pooled estimate was −17.8 µm (95% CI: −24.8 to −10.8; *p* < 0.001), confirming the primary result. Among proportion outcomes with 10 or more contributing studies, no significant asymmetry was detected for IRF (Peters *p* = 0.473) or SRF (Peters *p* = 0.439). Significant asymmetry was observed for IOI (Peters *p* = 0.002). Funnel plots are presented in Figure S7. A summary of all pooled outcomes and findings is presented in Table 3.

## 4. Discussion

This meta-analysis is the first to synthesize real-world outcomes after switching from prior anti-VEGF therapy to aflibercept 8 mg in previously treated nAMD. We combined data from 21 studies enrolling 6448 eyes, with co-primary efficacy pools of 1274 (BCVA), 1365 (CST), and 908 (interval) eyes. Switching maintained visual acuity (WMD −0.017 logMAR; 95% CI: −0.027 to −0.007; equivalent to +0.83 ETDRS letters) led to a significant reduction in central subfield thickness (WMD −21.5 µm; 95% CI: −29.3 to −13.7), and allowed for a longer treatment interval by an average of 1.79 weeks (95% CI: +1.32 to +2.27). Regarding safety, intraocular inflammation occurred in 0.2% of cases (95% CI: 0.0 to 1.0), and there were no confirmed cases of retinal vasculitis. Although there was considerable heterogeneity, sensitivity and leave-one-out analysis supported the reliability of these results.

### 4.1. Visual Acuity Stability

Stability in BCVA is expected in a population treated with a long prior duration of 15 to 60 anti-VEGF injections across studies [18,20,25,27,31,32]. The PULSAR trial showed gains of +6.7 and +6.2 letters at 48 weeks in treatment-naïve eyes [12]. In contrast, the smaller gains observed in patients who switched treatments seem to reflect the long-term nature of the disease, rather than a lack of drug effectiveness. Similarly, Spooner et al. [44] reported a pooled BCVA gain of just +1.11 letters (95% CI: −0.25 to +2.46; *p* = 0.17) in their meta-analysis of switching to aflibercept 2 mg, which showed a similar pattern of visual stability. The similarity between the aflibercept 2 mg and 8 mg switch eras in maintaining visual acuity suggests a functional limit for switching agents in this difficult patient group. However, the short follow-up period and varied baseline characteristics limit how strongly we can draw this conclusion. Additionally, a Bayesian network meta-analysis of treatment-naïve trial data similarly found no significant difference in BCVA between aflibercept 8 mg and faricimab (−1.14 letters; 95% CrI: −3.53 to +1.25) [45]. The pooled heterogeneity was absent for BCVA. However, the pooled estimate of the studies reported a change in ETDRS letter (WMD = +0.002; k = 7) which was not statistically significant. This implies that converting ETDRS to logMAR may add measurement imprecision rather than reflecting a real difference between reporting subgroups.

### 4.2. Central Subfield Thickness Reduction

The CST reduction was statistically significant and clinically meaningful, indicating that residual fluid persisting despite prior anti-VEGF therapy was further resolved. This follows the superior fluid-resolution results in the Phase 2 CANDELA trial [11] and the fourfold higher molar concentration. The aflibercept 2 mg switch meta-analysis by Spooner et al. [44] found CST reductions of −61.9 μm (95% CI: −77.1 to −46.8) at 6 months and −50.0 μm (95% CI: −63.2 to −36.8) at 12 months. These reductions are about three times larger than we found for three reasons: First, Spooner’s group switched only from bevacizumab or ranibizumab, a larger pharmacological change than switching from aflibercept 2 mg or faricimab predominantly in our cohort. Second, Spooner’s study required active exudative fluid at baseline, while up to 30% of eyes in some of our included studies were already anatomically dry at the time of switching [13,20]. Third, baseline CST was generally higher in Spooner’s group (up to 449 μm), and one included study found that higher baseline thickness predicted a greater CST reduction (−44.1 μm per 100-μm increase; *p* < 0.001) [13]. The faricimab switch meta-analysis by Zhang et al. [46], which included 10 studies with 721 eyes, reported a CST decrease of 46.67 µm (95% CI: 35.91 to 57.42; I^2^ = 0%), which is approximately double our estimate, likely because their absolute group mean difference yields larger effect sizes compared to our within-subject paired change scores and their cohort included eyes switching from a wider range of prior agents with potentially greater baseline fluid. Another switch-to-faricimab study by Sim et al. [47] included 106 eyes and reported a 12-month CST reduction of 22.1 µm (*p* = 0.041), which is close to our finding. Moreover, the network meta-analysis by Friedman et al. [45] included treatment-naïve groups comparing faricimab and aflibercept 8 mg and found that there was no significant difference in CST change between the two groups (mean difference: 1.17 µm; 95% CrI: −9.60 to +11.92); importantly aflibercept 8 mg needed fewer injections over 104 weeks (mean difference: −1.47; 95% CrI: −1.90 to −1.05). Our CST analysis showed moderate heterogeneity (I^2^ = 56.0%; Q = 38.68, df = 17, *p* = 0.002), which is expected because baseline CST varied across studies from 230.5 µm [24] to 371.6 µm [21], allowing more room for anatomical improvement. For all predefined sensitivity analyses, heterogeneity did not decrease significantly (residual I^2^ values ranged from 54.6% to 58.4%) (Table S6), suggesting other relevant factors, such as OCT measurement terms, retreatment rules, and fluid definitions. The funnel plot was borderline asymmetric (Egger *p* = 0.048), which may reflect small-study effects rather than real publication bias, since the CST reduction was consistent across the 18 included studies.

### 4.3. Treatment Interval Extension

Treatment interval extension, while modest in absolute magnitude, is a clinically relevant finding in this analysis. On average, intervals increased by 1.79 weeks (95% CI: +1.32 to +2.27; *p* < 0.001), which means patients needed 2 to 3 fewer injections each year. This helps reduce the injection burden that often leads to patients missing treatments. It also lowers procedural costs and makes the process less inconvenient. Similarly, Zhang et al. [46] found a comparable interval extension of 1.56 weeks (95% CI: 0.71 to 2.40; I^2^ = 86%; 591 eyes) in their meta-analysis of faricimab switches, showing both agents offer similar durability when switching. As expected, the intervals achieved were still shorter than those seen in the treatment-naïve PULSAR cohort [12]. In our analysis, the proportion of eyes successfully extended to maximum treat-and-extend intervals of ≥8, ≥12, and ≥16 weeks was 69.8% (k = 8), 13.7% (k = 6), and 9.6% (k = 4), respectively (Figure S3), compared with 83% of treatment-naïve patients who maintained 12-week intervals for 48 weeks [12]. Factors such as chronic neovascularization, treatment resistance, fibrotic changes, and shorter starting intervals explain these differences [48]. The heterogeneity for this outcome was substantial (I^2^ = 74.3%). Subgroup analysis according to the reasons for the switch partly explains the high heterogeneity (residual I^2^ = 54.5%). Refractory eyes gained more extension (+2.23 weeks [24,26]) compared to persistent but stable eyes (+1.16 weeks [16,18,31]). Additionally, switched eyes for only the purpose of interval extension gained a meaningful increase (+2.22 weeks [20,25,29]) (Table S7). Follow-up duration contributed to heterogeneity (residual I^2^ = 59.1%), with shorter follow-up periods yielding larger interval gains (+2.35 weeks for <6 months versus +1.53 weeks for ≥6 months), likely reflecting early loading-phase effects that lessen as treat-and-extend protocols reach equilibrium.

### 4.4. Secondary Anatomical Outcomes

Anatomical secondary outcomes further supported the efficacy of switching, with resolution of intraretinal fluid (IRF) and subretinal fluid (SRF) in 37.5% of eyes (95% CI: 27.7–48.5, k = 12; and 95% CI: 29.7–46.1, k = 11, respectively) (Figure S2). About one-third of eyes with persistent fluid at baseline achieved complete resolution after switching [13,15,16,18,19,20,21,23,24,25,26,31]. These fluid resolution rates are in line with real-world faricimab switch data. Grimaldi et al. [49] found that 38% of 353 eyes at 11 Swiss centers reported dryness following a single faricimab injection, a rate comparable to our pooled estimates, suggesting similar fluid clearance across newer anti-VEGF agents in chronically treated eyes. PED height also decreased by a pooled WMD of −22.9 µm (95% CI: −31.2 to −14.6; *p* < 0.001; k = 9; I^2^ = 35.4%) (Figure S4). This result was also stable across the prespecified correlation sensitivity analyses from r = 0.3 to r = 0.8 (range: −22.9 to −23.2 µm) (Supplementary Table S8). Taken together, the higher molar dose led to enhanced anatomical dryness. This is likely due to its greater VEGF-binding capacity and longer-lasting drug levels in the eye [11]. Importantly, these anatomical improvements occurred without visual acuity loss, which is relevant given that persistent fluid drives photoreceptor atrophy and long-term vision decline [50].

### 4.5. Safety Profile

The safety profile was reassuring, based on the largest outcome pool in this study. Including 20 studies and 9959 eyes, the incidence of IOI was 0.2% (95% CI: 0.0–1.0); 15 studies reported no IOI events [14,15,16,18,19,22,23,24,26,28,29,30,31,32,33], and no confirmed cases of retinal vasculitis. Compared with the pooled trial rate of 1.3% in CANDELA, PHOTON, and PULSAR [51], this difference is likely due to differences in adverse-event recording methods in real-world practice and shorter follow-up. This pooled trial analysis also reported comparable inflammation rates between aflibercept 8 mg and 2 mg [51], indicating that the higher dose does not increase inflammatory risk. Both rates are better than those observed with brolucizumab, which has been linked to retinal vasculitis and retinal vascular occlusion [52]. Rebsdorf et al. [53] reported an IOI incidence of 0.59% per injection (95% CI: 0.18 to 1.20) across 8834 aflibercept 8 mg injections, with a higher rate in real-world settings (0.93%) than in clinical trials (0.15%). Our per-eye estimate of 0.2% is not directly comparable to per-injection rates, though both are low. Rebsdorf et al. identified 12 cases from two Japanese cohorts. One of them was identified in our search but excluded (Matsumoto et al.) [54], because the outcomes were reported for a mixed cohort without separate switch-subgroup data. The significant funnel plot asymmetry for IOI (Peters *p* = 0.002) is due to the structure of rare-event data, as the two largest studies [17,27] accounted for most of the observed events. This reflects the statistical behavior of near-zero event rates rather than true publication bias.

### 4.6. Discontinuation Rates

The pooled discontinuation rate for all causes was 19.9% (95% CI: 13.3 to 28.7; k = 9; I^2^ = 86.0%) (Figure S5). The rate varied between 0% [32] and 41.3% [25] across different studies. Where studies explained the reasons for discontinuation, the most common cause was the inability to extend treatment intervals to the regulatory minimum of 8 weeks, particularly in jurisdictions such as the United States and Japan, where this minimum interval is mandated [16,25,26]. Although the subgroup analysis found similar discontinuation rates in places with (21.9%) and without (19.1%) required minimum intervals, the reasons for discontinuation differed. In label-constrained jurisdictions, discontinuation reflected failure to meet the 8-week minimum, whereas in non-constrained jurisdictions, it was driven by clinician-assessed inadequate response or patient preference. Stopping aflibercept 8 mg rarely meant stopping the anti-VEGF therapy altogether. In Kataoka et al. [25], the population that stopped taking this drug reverted to aflibercept 2 mg, which accounted for 94% of discontinued patients. In Bala et al. [16], 66.7% of patients who discontinued treatment switched to faricimab. These findings suggest that clinicians trialed aflibercept 8 mg for durability and reverted to established agents when targets were not met. The highest reported IOI incidence in a study was 3.9%, and all cases improved with topical corticosteroids [27]. Overall, the high discontinuation rate reflects regulatory, clinical, and patient factors rather than drug failure.

### 4.7. Limitations

The limitations of this review include: All the studies pooled were observational and used single-arm, pre-post designs without randomized comparison groups, so we cannot draw causal conclusions, which resulted in generally low GRADE scores. The certainty of evidence was rated low for BCVA and very low for CST and treatment interval (Supplementary Table S1), mainly because of study design and inconsistency. Second, there was a lot of variation in follow-up times, baseline characteristics, and dosing protocols across studies, which yielded less precise pooled estimates for CST (I^2^ = 56.0%) and treatment interval (I^2^ = 74.3%). Third, in most studies, within-subject standard deviations were estimated from cross-sectional data, but sensitivity analyses (r = 0.3–0.7) showed the results were robust. Fourth, few studies included both eyes from the same patient, which may slightly narrow the confidence intervals. Finally, most follow-up periods were less than 12 months, so longer-term data are still needed.

## 5. Conclusions

This is the first systematic review and meta-analysis to examine real-world switching to aflibercept 8 mg in patients who have already been treated with anti-VEGF therapy for nAMD. The results show that BCVA stayed stable, CST decreased, treatment intervals increased, and over one-third of eyes achieved fluid resolution. Extending the interval by almost two weeks, despite the magnitude varying by reason for switch, translates to two to three fewer injections each year. Intraocular inflammation was infrequent, with no confirmed retinal vasculitis, although the safety pool was not restricted to switched eyes. However, the certainty of evidence is low for BCVA and very low for CST and treatment interval, and these findings should be interpreted accordingly. Switching may be appropriate for persistent disease activity or high treatment burden, pending confirmation from longer-term comparative studies, particularly versus faricimab in the switch setting.

## Figures and Tables

**Table 1:** Characteristics of Included Studies.

Study	Country	Design	Eyes (*n*)	Age	Male (%)	Prior Injections	Prior Anti-VEGF Agent(s)	Loading	Regimen	FU (mo)	BCVA Unit	CST Term	OCT Device
**Abu Ishkheidem 2026** [13]	Sweden	Retrospective cohort	181	80.4 ± 8.5	32.3	Median 9 (IQR 2–57)	Aflib 2 mg 72.5%, Beva 12.4%, Fari 12.4%	No	T&E	3.0	logMAR	CRT	Topcon Triton
**Angus 2026** [14]	Australia	Retrospective cohort	42	80.5	42.9	NR (min. 3)	Aflib 2 mg 83.3%, Fari 14.3%, Rani 2.4%	No	T&E	9.6	ETDRS	CST	SD-OCT
**Bailey 2026** [15]	Global (18)	Prospective observational	104	79.5 ± 7.3	42.3	NR	Aflib 2 mg 53.9%, Fari 17.3%, Rani 14.4%	No	Local practice	2.0	ETDRS	CRT	Site-specific
**Bala 2025** [16]	USA	Retrospective cohort	219	79.6 ± 7.9	44.0	22.6 ± 23.6	Aflib 2 mg 57.1%, Fari 26%, Beva 11%	No	T&E	5.7	ETDRS	CST	Zeiss Meditec
**Bates 2025** [17]	USA	Retrospective multicenter	4823	NR	45.2	6.6 ± 2.8 (12 mo)	NR	No	T&E/PRN	2.8	logMAR	NR	NR
**Cheung 2026** [18]	Singapore	Retrospective audit	30	71.3 ± 7.1	55.2	27.6 ± 19.3	Aflib 2 mg 76.7%, Fari 23.3%	No	T&E	6.8	logMAR	CRT	NR
**Chi 2025** [19]	Taiwan	Retrospective case series	20	69 (median)	70.0	NR (min. 3)	Aflib 2 mg 60%, Fari 40%	No	PRN-like	3.0	logMAR	CMT	OptoVue Avanti
**Emfietzoglou 2026** [20]	Greece	Retrospective case series	74	80.0 (median)	50.0	Median 15 (IQR 9–24)	Aflib 2 mg 90.5%, Beva 9.5%	No	T&E	4.0	logMAR	CFT	Optovue Avanti
**Grün 2025** [21]	Germany	Retrospective	51	82.0 ± 7.3	39.3	Mean 39 (3–141)	Aflib 2 mg 66.7%, Fari 25.5%, Rani 3.9%	Yes	T&E	4.0	logMAR	CSRT	Spectralis
**Hafner 2025** [22]	Germany	Retrospective	23	78.9 ± 7.4	19.0	30.7 ± 22.5	Aflib 2 mg 100%	No	T&E	3.7	logMAR	CRT	Spectralis
**Hara 2025** [23]	Japan	Retrospective observational	102	79.6 ± 8.0	62.2	30.8 ± 22.1	Aflib 2 mg 89.2%, Fari 4.9%, Brolu 5.9%	No	PRN-like (Interval matched)	2.1	logMAR	CFT	Zeiss/Topcon
**Janmohamed 2026** [24]	UK	Retrospective cohort	66	79.5 (median)	48.3	Median 17 (IQR 12–36.5)	Fari 65.2%, Aflib 2 mg 16.7%, Rani 18.2%	No	T&E	6.0	ETDRS	CMT	Topcon Triton
**Kataoka 2026** [25]	Japan	Multicenter retrospective	121	77.0 ± 8.8	73.5	Median 31 (IQR 20.5–55.5)	Aflib 2 mg 81%, Fari 14%, Brolu 2%	No	T&E	6.0	logMAR	CRT	Topcon/Spectralis
**Kindo 2026** [26]	Japan	Retrospective observational	44	76.5 (median)	65.9	Median 28 (IQR 7–50)	Aflib 2 mg 100%	No	T&E	6.0	logMAR	CRT	Topcon/Spectralis
**Kitay 2026** [27]	Switzerland	Multicenter retrospective	246	81.0 (median)	33.9	33.0 ± 27.6	Aflib 2 mg 70.3%, Fari 25.8%, Rani 3.9%	No	T&E	12.0	ETDRS	CST	Topcon/Spectralis
**Mizukami 2025** [28]	Japan	Retrospective observational	15	79.7 ± 7.3	61.5	12.1 ± 7.1	Aflib 2 mg 66.7%, Brolu 20%, Fari 13.3%	No	PRN-like (Interval matched)	1.0	logMAR	CMT	Topcon Triton
**Mizuma 2026** [29]	Japan	Multicenter retrospective	35	78.7 ± 9.4	52.5	Median 10 (IQR 5.8–19.3)	Aflib 2 mg 100%	Yes	T&E	12.0	logMAR	CST	Site-specific
**Momenaei 2025** [30]	USA	Retrospective chart review	135	80.6 ± 8.1	51.9	7.4 ± 4 (fari)	Fari 100%, Aflib 80%, Beva 40%	No	T&E	NR	ETDRS	CFT	Spectralis
**Musadiq 2025** [31]	UK	Retrospective observational	59	80.2 ± 6.3	56.0	26.9 ± 19.0	Fari 59.3%, Aflib 2 mg 37.3%, Brolu 3.4%	No	T&E	7.7	ETDRS	CST	Spectralis
**Palm 2025** [32]	Switzerland	Retrospective single-arm	22	77.1 ± 7.6	55.6	Median 60 (IQR 16–77.8)	Aflib 2 mg 59.1%, Rani 13.6%, Fari 27.3%	No	T&E	3.8	ETDRS	CST	Spectralis SD-OCT
**Sambhara 2025** [33]	USA	Retrospective case series	36	79.8	47.5	35.5 (3–98)	Fari 55.6%, Aflib 2 mg 41.7%, Beva 2.7%	No	Unstandardized	6.0+	logMAR	CST	NR

Abbreviations: Aflib, aflibercept; Beva, bevacizumab; Brolu, brolucizumab; CFT, central foveal thickness; CMT, central macular thickness; CRT, central retinal thickness; CST, central subfield thickness; CSRT, central subretinal thickness; ETDRS, Early Treatment Diabetic Retinopathy Study; Fari, faricimab; FU, follow-up; mo, months; NR, not reported; PRN, pro re nata; Rani, ranibizumab; SD-OCT, spectral-domain optical coherence tomography; T&E, treat-and-extend. OCT device manufacturers: Topcon (Topcon Corporation, Tokyo, Japan); Spectralis (Heidelberg Engineering, Heidelberg, Germany); Zeiss (Carl Zeiss Meditec, Jena, Germany); Optovue (Optovue Inc., Fremont, CA, USA).

**Table 2:** Baseline Clinical Characteristics.

Study	Baseline BCVA	Baseline CST (µm)	Pre-Switch Interval (wk)	nAMD Subtype	Disease Duration	Reason for Switch
**Abu Ishkheidem 2026** [13]	0.46 ± 0.31	249.4 ± 67.7	NR	NR	NR	Mixed
**Angus 2026** [14]	68	326	7.4	NR	3.1 years	Mixed
**Bailey 2026** [15]	61.6 ± 19.4	316 ± 102	NR	Type 1 (27.9%), Type 2 (10.6%), Type 3 (4.8%), Unknown (56.7%)	36.9 ^†^ mo	Clinician decision
**Bala 2025** [16]	61.9 ± 21.7	267.8 ± 72.5	5.8 ± 2.5	NR	NR	Persistent/durability
**Bates 2025** [17]	0.48	NR	5.4	NR	NR	Persistent/extension
**Cheung 2026** [18]	0.32 [0.18–0.50]	291 [275–301]	8 [7–10]	Type 1 MNV (50%), PCV (36.7%), Type 2 MNV (6.7%), Mixed (6.7%)	60.4 ± 42.7 mo	Suboptimal/extension
**Chi 2025** [19]	0.52 [0.40–0.92]	304.50 [241.50–364.75]	NR	nAMD (60%), PCV (25%), PNV (15%)	NR	Refractory
**Emfietzoglou 2026** [20]	0.40 [0.20–0.60]	283.0 [201.2–406.2]	10.0 [7.25–12.0]	Type 1 48.6%, fibrovascular PED 27.0%, PCV 10.8%, Pachy MNV T1 5.4%, Mixed 4.1%, Type 3 2.7%, Type 2 1.4%	3.2 (2.0–7.0) years	Durability-limited
**Grün 2025** [21]	0.48 ± 0.32	371.6 ± 120.7	NR	NR	NR	Insufficient effect
**Hafner 2025** [22]	0.20 [0.40]	335.6 ± 136.9	4.86	NR	3.49 ± 2.98 years	Refractory
**Hara 2025** [23]	0.27 ± 0.35	289 ± 115	8.9 ± 2.8	PCV (44.1%), Type 1 (47.1%), Type 2 (2.9%), Type 3 (5.9%)	6.0 ± 4.0 years	Exudative changes
**Janmohamed 2026** [24]	70	230.5	4	Atrophy 86.4%, Fibrosis 40.9%	4.0 years	Inadequate response
**Kataoka 2026** [25]	0.15 [0.00–0.30]	258 [203–329]	6.0 [5.0–8.0]	Type 1 MNV 68%, PCV 30%, Type 2 1%, Type 3 2%	NR	Frequent injections
**Kindo 2026** [26]	0.05 [−0.08–0.22]	287.0 [213.0–373.0]	7.0 [6.0–9.0]	Type 1 47.7%, PCV 47.7%, Type 2 4.5%	NR	Extension failure
**Kitay 2026** [27]	69.5 ± 15.7	329.1 ± 91.3	7.1 ± 2.7	Type 1 59.0%, Type 2 15.9%, Mixed 5.3%, Type 3 6.4%, PCV 3.5%	4.1 ± 3.2 years	Persistent/extension
**Mizukami 2025** [28]	0.46 ± 0.57	290.40 ± 82.55	21.3 ± 20.9	Type 1 80%, Type 2 6.7%, PCV 13.3%	NR	Insufficient response
**Mizuma 2026** [29]	NR	NR	8 [7–12]	Type 1 40%, Type 2 42.5%, PCV 17.5%	NR	Durability improvement
**Momenaei 2025** [30]	63.9 ± 14.4	325 ± 104	7.57	NR	NR	Suboptimal to Fari
**Musadiq 2025** [31]	66.0 ± 14.4	367.2 ± 100.7	7.7 ± 1.7	NR	NR	Persistent/extension
**Palm 2025** [32]	76 [70.8–79.0]	280.0 ± 53.4	5.5 [5.0–8.0]	Type 1 86.4%, Mixed 13.6%	NR	Extension goal
**Sambhara 2025** [33]	0.307 ± 0.24	328.9 ± 94.2	5.86	NR	NR	Recalcitrant

^†^ Median reported. Values are presented as mean ± SD, median (IQR), or *n* (%) as reported in the original studies. Abbreviations; BCVA, best-corrected visual acuity; CST, central subfield thickness; Fari, faricimab; IQR, interquartile range; MNV, macular neovascularization; mo, months; NR, not reported; PCV, polypoidal choroidal vasculopathy; PED, pigment epithelial detachment; PNV, pachychoroid neovasculopathy; SD, standard deviation; wk, weeks.

**Table 3:** Summary of Findings.

Outcome	k	Total Eyes	Method	Pooled Estimate [95% CI]	*p*	I^2^	Q *p*	Certainty (GRADE)
**Co-primary outcomes**								
BCVA change (logMAR)	18 ^†^	1274	DL + HKSJ	−0.017 [−0.027, −0.007]	0.002	0.0%	0.628	Low ⊕⊕◯◯ ^††^
ETDRS equivalent				+0.83 letters [+0.34, +1.32]				
CST change (µm)	18	1365	DL + HKSJ	−21.5 [−29.3, −13.7]	<0.001	56.0%	0.002	Very low ⊕◯◯◯ ^††^
Interval change (wk)	10	908	DL + HKSJ	+1.79 [+1.32, +2.27]	<0.001	74.3%	<0.001	Very low ⊕◯◯◯ ^††^
**Anatomical outcomes**								
IRF resolution	12	470	GLMM	37.5% [27.7, 48.5]	0.026	75.9%	<0.001	—
SRF resolution	11	595	GLMM	37.5% [29.7, 46.1]	0.005	73.0%	<0.001	—
PED height change (µm)	9	729	DL + HKSJ	−22.9 [−31.2, −14.6]	<0.001	35.4%	0.135	—
**Durability outcomes**								
≥8-week interval achievement	8	868	GLMM	69.8% [43.2, 87.6]	0.140	97.9%	<0.001	—
≥12-week interval achievement	6	549	GLMM	13.7% [5.9, 28.8]	<0.001	89.5%	<0.001	—
≥16-week interval achievement	4	493	GLMM	9.6% [3.6, 23.1]	<0.001	89.9%	<0.001	—
**Safety outcomes**								
IOI incidence	20	9959	GLMM	0.2% [0.0, 1.0]	<0.001	81.6%	<0.001	—
Discontinuation rate	9	945	GLMM	19.9% [13.3, 28.7]	<0.001	86.0%	<0.001	—

^†^ Of 18 studies pooled for BCVA, 10 required SD imputation (r = 0.5), 6 reported natively, 1 CI-derived, and 1 Wan-converted. Similar proportions applied to CST (12 of 18 imputed) and treatment interval (6 of 10 imputed). ^††^ GRADE certainty of evidence: ⊕⊕⊕⊕ high, ⊕⊕⊕◯ moderate, ⊕⊕◯◯ low, ⊕◯◯◯ very low. Full GRADE assessment is provided in Supplementary Table S1. Abbreviation: BCVA, best-corrected visual acuity; CST, central subfield thickness; PED, pigment epithelial detachment; IRF, intraretinal fluid; SRF, subretinal fluid; IOI, intraocular inflammation; GLMM, generalized linear mixed model with logit link; DL + HKSJ, DerSimonian–Laird estimator with Hartung–Knapp–Sidik–Jonkman adjustment. Co-primary outcomes are reported as weighted mean difference; proportion outcomes are reported as pooled percentages with 95% confidence intervals.

## Data Availability

Data are available upon reasonable request to the corresponding author.

## Supplementary Materials

The following supporting information can be downloaded at: https://www.mdpi.com/article/10.3390/jcm15124599/s1, including search strings, Table S1. GRADE certainty of evidence assessment for co-primary outcomes. Table S2. Reasons for exclusions at the full-text screening phase. Table S3. Comparison of GLMM-logit and Freeman–Tukey pooled estimates for proportion outcomes. Table S4. Sensitivity analyses for co-primary outcomes. Table S5. Subgroup analyses for BCVA (logMAR). Table S6. Subgroup analyses for CST (µm). Table S7. Subgroup analyses for treatment interval (weeks). Table S8. Correlation sensitivity analysis for PED height change. Figure S1. Risk of bias assessment of included studies according to the JBI Critical Appraisal Checklist for Case Series. Figure S2. Forest plots of pooled (A) intraretinal fluid (IRF) and (B) subretinal fluid (SRF) resolution rates after switching to aflibercept 8 mg. The pooled proportions displayed reflect the Freeman–Tukey double-arcsine transformation; the primary GLMM-logit estimates are reported in the text. Figure S3. Forest plots of pooled proportions of eyes achieving treatment intervals of (A) ≥8 weeks, (B) ≥12 weeks, and (C) ≥16 weeks after switching to aflibercept 8 mg. Pooled proportions were estimated using the Freeman–Tukey double-arcsine transformation with DerSimonian–Laird estimation. Figure S4. Forest plot of pooled change in pigment epithelial detachment (PED) height (µm) after switching to aflibercept 8 mg, estimated using a random-effects model (DerSimonian–Laird estimator with Hartung–Knapp–Sidik–Jonkman adjustment). Figure S5. Forest plot of pooled discontinuation rate after switching to aflibercept 8 mg, estimated using the Freeman–Tukey double-arcsine transformation with DerSimonian–Laird estimation. Figure S6. Leave-one-out sensitivity analyses for co-primary outcomes. Each row shows the pooled estimate after excluding the named study; vertical reference lines indicate the primary pooled estimates. (A) BCVA change, reference −0.017 logMAR; (B) CST change, reference −21.5 µm; (C) treatment interval change, reference +1.79 weeks. Figure S7. Funnel plots for publication bias assessment. (A) BCVA change, Egger p = 0.771; (B) CST change, Egger p = 0.048; (C) treatment interval change, Egger p = 0.783; (D) IOI incidence, Peters p = 0.002; (E) IRF resolution, Peters p = 0.473; (F) SRF resolution, Peters p = 0.439.and the PRISMA 2020 Checklist.

## Abbreviations

The following abbreviations are used in this manuscript:

nAMD	Neovascular Age-Related Macular Degeneration
anti-VEGF	Anti-Vascular Endothelial Growth Factor
VEGF	Vascular Endothelial Growth Factor
BCVA	Best-Corrected Visual Acuity
CST	Central Subfield Thickness
IRF	Intraretinal Fluid
SRF	Subretinal Fluid
PED	Pigment Epithelial Detachment
IOI	Intraocular Inflammation
logMAR	Logarithm of the Minimum Angle of Resolution
ETDRS	Early Treatment Diabetic Retinopathy Study
WMD	Weighted Mean Difference
CI	Confidence Interval
DL	DerSimonian–Laird
HKSJ	Hartung–Knapp–Sidik–Jonkman
GLMM	Generalized Linear Mixed Model
GRADE	Grading of Recommendations, Assessment, Development and Evaluation
PRISMA	Preferred Reporting Items for Systematic Reviews and Meta-Analyses
PROSPERO	International Prospective Register of Systematic Reviews
JBI	Joanna Briggs Institute
OCT	Optical Coherence Tomography
MeSH	Medical Subject Headings
CrI	Credible Interval
MNV	Macular Neovascularization
PCV	Polypoidal Choroidal Vasculopathy
T&E	Treat-and-Extend
PRN	Pro Re Nata
IQR	Interquartile Range

## References

[1] Wong W.L., Su X., Li X., Cheung C.M.G., Klein R., Cheng C.-Y., Wong T.Y. (2014). Global Prevalence of Age-Related Macular Degeneration and Disease Burden Projection for 2020 and 2040: A Systematic Review and Meta-Analysis. Lancet Glob. Health.

[2] Mitchell P., Liew G., Gopinath B., Wong T.Y. (2018). Age-Related Macular Degeneration. Lancet.

[3] Rosenfeld P.J., Brown D.M., Heier J.S., Boyer D.S., Kaiser P.K., Chung C.Y., Kim R.Y., MARINA Study Group (2006). Ranibizumab for Neovascular Age-Related Macular Degeneration. N. Engl. J. Med..

[4] Martin D.F., Maguire M.G., Fine S.L., Ying G.S., Jaffe G.J., Grunwald J.E., Toth C., Redford M., Ferris F.L., Comparison of Age-Related Macular Degeneration Treatments Trials (CATT) Research Group (2012). Ranibizumab and Bevacizumab for Treatment of Neovascular Age-Related Macular Degeneration: Two-Year Results. Ophthalmology.

[5] Heier J.S., Brown D.M., Chong V., Korobelnik J.-F., Kaiser P.K., Nguyen Q.D., Kirchhof B., Ho A., Ogura Y., Yancopoulos G.D. (2012). Intravitreal Aflibercept (VEGF Trap-Eye) in Wet Age-Related Macular Degeneration. Ophthalmology.

[6] Dugel P.U., Singh R.P., Koh A., Ogura Y., Weissgerber G., Gedif K., Jaffe G.J., Tadayoni R., Schmidt-Erfurth U., Holz F.G. (2021). HAWK and HARRIER: Ninety-Six-Week Outcomes from the Phase 3 Trials of Brolucizumab for Neovascular Age-Related Macular Degeneration. Ophthalmology.

[7] Heier J.S., Khanani A.M., Quezada Ruiz C., Basu K., Ferrone P.J., Brittain C., Figueroa M.S., Lin H., Holz F.G., Patel V. (2022). Efficacy, Durability, and Safety of Intravitreal Faricimab up to Every 16 Weeks for Neovascular Age-Related Macular Degeneration (TENAYA and LUCERNE): Two Randomised, Double-Masked, Phase 3, Non-Inferiority Trials. Lancet.

[8] Holz F.G., Tadayoni R., Beatty S., Berger A., Cereda M.G., Cortez R., Hoyng C.B., Hykin P., Staurenghi G., Heldner S. (2015). Multi-Country Real-Life Experience of Anti-Vascular Endothelial Growth Factor Therapy for Wet Age-Related Macular Degeneration. Br. J. Ophthalmol..

[9] Yap D.W.T., Tan B.K.J., Chong K.T.Y., Wong T.Y., Cheung C.M.G. (2025). Persistence of Retinal Fluid after Anti-VEGF Treatment for Neovascular Age-Related Macular Degeneration: A Systematic Review and Meta-Analysis. Ophthalmol. Retin..

[10] Rofagha S., Bhisitkul R.B., Boyer D.S., Sadda S.R., Zhang K. (2013). Seven-Year Outcomes in Ranibizumab-Treated Patients in ANCHOR, MARINA, and HORIZON. Ophthalmology.

[11] Wykoff C.C., Brown D.M., Reed K., Berliner A.J., Gerstenblith A.T., Breazna A., Yau L., Tian Y., Bourcier M.E., Boyer D.S. (2023). Effect of High-Dose Intravitreal Aflibercept, 8 mg, in Patients with Neovascular Age-Related Macular Degeneration: The Phase 2 CANDELA Randomized Clinical Trial. JAMA Ophthalmol..

[12] Lanzetta P., Korobelnik J.-F., Heier J.S., Leal S., Holz F.G., Clark W.L., Ehlken C., Bressler N.M., Silverman D., Henry E. (2024). Intravitreal Aflibercept 8 mg in Neovascular Age-Related Macular Degeneration (PULSAR): 48-Week Results from a Randomised, Double-Masked, Non-Inferiority, Phase 3 Trial. Lancet.

[13] Abu Ishkheidem I., Inci E., Breimer M., Silfverswärd S.T., Zetterberg M., Grönlund M.A. (2026). Real-World Outcomes of Aflibercept 8 mg in Patients Previously Treated for Neovascular Age-Related Macular Degeneration. Acta Ophthalmol..

[14] Angus Z.G., Lee Y., Sandhu S.S., Troutbeck R., Yeoh J., Chiu D., Lim L.L. (2026). Switching to Faricimab or High-Dose Aflibercept for Neovascular AMD in High-Demand Patients: Impact on Injection Interval in a Real-World Cohort. Clin. Exp. Ophthalmol..

[15] Bailey C., Lange C., Chaudhary V., Lanzetta P., Oubraham H., Kirchner M., Berliner A.J., Loo J., Gruben D. (2026). SPECTRUM: Early Clinical Experience from the First Global Real-World Study of Aflibercept 8 mg in Patients with Neovascular Age-Related Macular Degeneration. Eye.

[16] Bala S., Barbosa G.C.S., Mohan N., Srivastava S.K., Kaiser P.K., Sastry A., Babiuch A.S., Singh R.P. (2025). Initial Functional and Anatomical Outcomes of High-Dose Aflibercept 8 mg in Exudative Neovascular Age-Related Macular Degeneration. Ophthalmol. Retin..

[17] Bates B.A., Mansour H.A., Al-Khersan H., Wood E., Momenaei B., Schneider E., Richards C.J., DeYoung C., Wykoff C.C., Quinn K. (2025). The Efficacy and Safety of Intravitreal Aflibercept 8 mg in Clinical Practice. J. Vitreoretin. Dis..

[18] Cheung C.M.G., Kikushima W., Teo K.Y.C. (2026). Initial Experiences of Switching to Aflibercept 8 mg for Neovascular Age-Related Macular Degeneration and Polypoidal Choroidal Vasculopathy in an Asian Population. Ophthalmol. Ther..

[19] Chi S.-C., Hwang D.-K., Chen S.-J., Weng C.-C., Huang Y.M., Chou Y.-B., Wu W.-C. (2026). Short-Term Efficacy of Aflibercept 8 mg in Refractory Neovascular Age-Related Macular Degeneration: A Retrospective Case Series from Taiwan. BMC Ophthalmol..

[20] Emfietzoglou M., Frances S., Charonis A. (2026). Early Real-World Anatomic Response and Interval Extension after Switching to Aflibercept 8 mg in Previously Treated Eyes with Neovascular AMD or Pachychoroid-Related Macular Neovascularization. AJO Int..

[21] Grün M., Rothaus K., Lommatzsch A.P., Faatz H. (2025). Early Outcome of Aflibercept 8 mg for Neovascular AMD in the Real-World Setting. Klin. Monbl. Augenheilkd..

[22] Hafner M., Asani B., Eckardt F., Liesenhoff C., Kufner A., Siedlecki J., Priglinger S.G., Wolf A. (2025). Deep Learning-Assisted Analysis of Biomarker Changes after Increase of Dosing from Aflibercept 2 mg to 8 mg in Therapy-Resistant Neovascular Age-Related Macular Degeneration. BMJ Open Ophthalmol..

[23] Hara C., Fujimoto S., Fukushima Y., Sayanagi K., Nishida K., Maruyama K., Nishida K. (2025). Initial Response after Switching to Aflibercept 8 mg for Neovascular Age-Related Macular Degeneration. J. Clin. Med..

[24] Janmohamed I.K., Harahn H., Carr B., Sahbi S., Ahmed H., Farahat A., Sherif M., Sherif S. (2026). Early Real-World Outcomes of Aflibercept 8 mg in Previously Treated Neovascular Age-Related Macular Degeneration. Eye.

[25] Kataoka K., Tanaka K., Honjyo J., Miyara Y., Hashiya N., Watanabe Y., Nishiguchi K.M., Kaneko H. (2026). Treatment Outcomes of Switching to Aflibercept 8 mg in Previously-Treated Neovascular Age-Related Macular Degeneration. Graefes Arch. Clin. Exp. Ophthalmol..

[26] Kindo H., Hosokawa M.M., Ouchi C., Matoba R., Morita T., Hayashi J., Kimura S., Morizane Y. (2026). Real-World Six-Month Outcomes after Switching from Aflibercept 2 mg to Aflibercept 8 mg for Neovascular Age-Related Macular Degeneration. Jpn. J. Ophthalmol..

[27] Kitay A.M., Tillmann A., Pfister I., Schild C., Bonanata M., Heck K., Ebneter A., Hatz K., Zinkernagel M.S. (2026). One-Year Real-World Outcomes of Switching to Aflibercept 8 mg in Eyes with Neovascular Age-Related Macular Degeneration: A Swiss Retina Research Network Report. Ophthalmol. Ther..

[28] Mizukami T., Ueno S., Mishima S., Shimomura Y. (2025). One-Month Real-World Comparison of Aflibercept 8 mg Versus 2 mg in Treatment-Naïve and Previously Treated Eyes with Neovascular Age-Related Macular Degeneration. Biologics.

[29] Mizuma R., Mizuki Y., Kamata A., Onishi J., Sakono T., Suzuki M., Terasaki H., Sonoda S. (2026). One-Year Real-World Outcomes of Intravitreal Aflibercept 8 mg for Neovascular Age-Related Macular Degeneration in Japan: A Multicenter Retrospective Study. Clin. Ophthalmol..

[30] Momenaei B., Yonekawa Y., Abril P., McCullough R., Abbey A.M. (2025). Outcomes of Intravitreal Aflibercept 8 mg in Eyes with Neovascular Age-Related Macular Degeneration Previously Treated with Faricimab. Ophthalmic Surg. Lasers Imaging Retin..

[31] Musadiq M., Musadiq M., Latif F., Ng B., Azzopardi M., Gilead N., Needham A., Chong Y.J. (2025). Early Real-World Outcomes of Switching to 8 mg Aflibercept for Neovascular Age-Related Macular Degeneration in the United Kingdom. Life.

[32] Palm C., Zweifel S.A., Gabathuler F., Cozzi M., Fasler K. (2025). Efficacy of Aflibercept 8 mg in Pretreated Age-Related Macular Degeneration. J. Clin. Med..

[33] Sambhara D., Vakharia P., Eichenbaum D.A. (2025). Real-World Efficacy and Safety of 8 mg Aflibercept in Neovascular AMD: A Case Series. BMJ Open Ophthalmol..

[34] Page M.J., McKenzie J.E., Bossuyt P.M., Boutron I., Hoffmann T.C., Mulrow C.D., Shamseer L., Tetzlaff J.M., Akl E.A., Brennan S.E. (2021). The PRISMA 2020 Statement: An Updated Guideline for Reporting Systematic Reviews. BMJ.

[35] Higgins J.P.T., Thomas J., Chandler J., Cumpston M., Li T., Page M.J., Welch V.A. (2022). Cochrane Handbook for Systematic Reviews of Interventions.

[36] Gregori N.Z., Feuer W., Rosenfeld P.J. (2010). Novel Method for Analyzing Snellen Visual Acuity Measurements. Retina.

[37] Wan X., Wang W., Liu J., Tong T. (2014). Estimating the Sample Mean and Standard Deviation from the Sample Size, Median, Range and/or Interquartile Range. BMC Med. Res. Methodol..

[38] Luo D., Wan X., Liu J., Tong T. (2018). Optimally Estimating the Sample Mean from the Sample Size, Median, Mid-Range, and/or Mid-Quartile Range. Stat. Methods Med. Res..

[39] Munn Z., Barker T.H., Moola S., Tufanaru C., Stern C., McArthur A., Stephenson M., Aromataris E. (2020). Methodological Quality of Case Series Studies: An Introduction to the JBI Critical Appraisal Tool. JBI Evid. Synth..

[40] Viechtbauer W. (2010). Conducting Meta-Analyses in R with the metafor Package. J. Stat. Softw..

[41] DerSimonian R., Laird N. (1986). Meta-Analysis in Clinical Trials. Control. Clin. Trials.

[42] Hartung J., Knapp G. (2001). A Refined Method for the Meta-Analysis of Controlled Clinical Trials with Binary Outcome. Stat. Med..

[43] Freeman M.F., Tukey J.W. (1950). Transformations Related to the Angular and the Square Root. Ann. Math. Stat..

[44] Spooner K., Hong T., Wijeyakumar W., Chang A.A. (2017). Switching to Aflibercept among Patients with Treatment-Resistant Neovascular Age-Related Macular Degeneration: A Systematic Review with Meta-Analysis. Clin. Ophthalmol..

[45] Friedman S.M., Xu Y., Sherman S., Kuznik A., Mojebi A., Keeping S., Bhandari A. (2025). Aflibercept 8 mg versus Faricimab Treat-and-Extend for Diabetic Macular Edema or Neovascular Age-Related Macular Degeneration: A Bayesian Fixed-Effect Network Meta-Analysis of Clinical Trials. Ophthalmol. Ther..

[46] Zhang C., Aboukasm G., Lai D.A., Leung N., Zhu D., Albini T.A., Berrocal A.M., Davis J.L., Flynn H.W., Gregori N.Z. (2025). Clinical Efficacy of Switching to Faricimab in Treatment-Resistant Neovascular Age-Related Macular Degeneration: Systematic Review and Meta-Analysis. Am. J. Ophthalmol..

[47] Sim S.Y., Chalkiadaki E., Koutsocheras G., Nicholson L., Sivaprasad S., Patel P.J., Tufail A., Balaskas K. (2025). Real-World 1-Year Outcomes of Treatment-Intensive Neovascular Age-Related Macular Degeneration Switched to Faricimab. Ophthalmol. Retin..

[48] Mettu P.S., Allingham M.J., Cousins S.W. (2021). Incomplete Response to Anti-VEGF Therapy in Neovascular AMD: Exploring Disease Mechanisms and Therapeutic Opportunities. Prog. Retin. Eye Res..

[49] Grimaldi G., Ambresin A., Pfister I.B., Schild C., Plasencia C., Hatz K., Ebneter A., Zinkernagel M.S. (2025). One-Year Outcomes after Switching to Faricimab in Eyes with Pretreated Neovascular Age-Related Macular Degeneration. Ophthalmol. Retin..

[50] Core J.Q., Pistilli M., Hua P., Daniel E., Grunwald J.E., Toth C.A., Jaffe G.J., Martin D.F., Maguire M.G., Ying G.S. (2022). Predominantly Persistent Intraretinal Fluid in the Comparison of Age-Related Macular Degeneration Treatments Trials. Ophthalmol. Retin..

[51] Stahl A., Schneider E., Schmidt-Ott U.M., Tueckmantel C., Schulze A., Berliner A.J., Chu K.W., Vitti R., Reed K., Zhang X. (2024). Pooled Safety Analysis of the CANDELA, PHOTON, and PULSAR Trials up to 96 Weeks Demonstrates Comparable Safety Profiles with Aflibercept 8 mg and 2 mg. Investig. Ophthalmol. Vis. Sci..

[52] Baumal C.R., Spaide R.F., Vajzovic L., Freund K.B., Walter S.D., John V., Rich R.M., Grossi F., Souied E.H., Fraunfelder F.W. (2020). Retinal Vasculitis and Intraocular Inflammation after Intravitreal Injection of Brolucizumab. Ophthalmology.

[53] Rebsdorf N.S., Michaelsen M.M., Ølholm N.K., Olsen S., Lee C., Subhi Y., Sørensen T.L. (2025). Inflammation and Increased Intraocular Pressure Associated with Intravitreal Aflibercept 8 mg: A Systematic Review and Meta-Analysis. AJO Int..

[54] Matsumoto H., Hoshino J., Numaga S., Mimura K., Asatori Y., Akiyama H. (2024). Retinal Vasculitis after Intravitreal Aflibercept 8 mg for Neovascular Age-Related Macular Degeneration. Jpn. J. Ophthalmol..

