# Supplementary material for: Real-World Outcomes of Switching to Aflibercept 8 mg in Previously Treated Neovascular Age-Related Macular Degeneration: A Systematic Review and Meta-Analysis
Source: J Clin Med. 2026 Jun 13;15(12):4599. doi: 10.3390/jcm15124599 (PMC13301121; doi:10.3390/jcm15124599)
Supplement: Supplementary file 1 [file jcm-15-04599-s001.zip › jcm-4369176-supplementary.pdf]

Supplementary Material for: Real-World Outcomes of Switching to Aflibercept 8 mg in Previously Treated Neovascular Age-Related Macular Degeneration: A Systematic Review and Meta-Analysis

Table of Contents

Content	Page
**Search Strings**	
PubMed/Medline & Web of Science	3
Cochrane CENTRAL & Scopus	4
Embase & ClinicalTrials.gov & Google Scholar	5
**Supplementary Tables**	
Table S1. GRADE Certainty of Evidence Assessment	6
Table S2. Reasons for Exclusions at Full-Text Screen Phase.	7-10
Table S3. GLMM-Logit vs Freeman-Tukey Comparison	11
Table S4. Sensitivity Analyses for Co-Primary Outcomes	11
Table S5. Subgroup Analyses for BCVA	12
Table S6. Subgroup Analyses for CST	13
Table S7. Subgroup Analyses for Treatment Interval	14
Table S8. Correlation Sensitivity Analysis for PED Height Change	15
**Studies Excluded from Quantitative Pooling**	16
**Supplementary Figures**	
Figure S1. JBI Risk of Bias assessment	17
Figure S2. Forest plot - IRF and SRF resolution	18
Figure S3. Forest plots - Proportion for ≥8 weeks, ≥12 weeks, ≥16 weeks achievements	19
Figure S4. Forest plot - PED height change	20
Figure S5. Forest plot - Discontinuation rate	20
Figure S6. Leave-one-out analyses	21
Figure S7. Funnel plots	22
**PRISMA 2020 Checklist**	23-25

Search was conducted on 4/24/2026

	**Pubmed/Medline**	**Total**
**#1**	"aflibercept 8 mg"[tiab] OR "aflibercept 8mg"[tiab] OR "aflibercept 8-mg"[tiab] OR "high-dose aflibercept"[tiab] OR "high dose aflibercept"[tiab] OR "Eylea HD"[tiab]	**120**
**#2**	"Macular Degeneration"[MeSH] OR "Wet Macular Degeneration"[MeSH] OR "Choroidal Neovascularization"[MeSH] OR "macular degeneration"[tiab] OR "neovascular age-related macular degeneration"[tiab] OR "neovascular age related macular degeneration"[tiab] OR "neovascular AMD"[tiab] OR "nAMD"[tiab] OR "wAMD"[tiab] OR "AMD"[tiab] OR "ARMD"[tiab] OR "wet AMD"[tiab] OR "wet age-related macular degeneration"[tiab] OR "wet age related macular degeneration"[tiab] OR "wet macular degeneration"[tiab] OR "exudative AMD"[tiab] OR "exudative age-related macular degeneration"[tiab] OR "exudative macular degeneration"[tiab] OR "choroidal neovascularization"[tiab] OR "choroidal neovascularisation"[tiab] OR "macular neovascularization"[tiab] OR "macular neovascularisation"[tiab] OR "CNV"[tiab] OR "CNVM"[tiab] OR "MNV"[tiab] OR "age-related maculopath*"[tiab] OR "age related maculopath*"[tiab] OR "retinal angiomatous proliferation"[tiab] OR "polypoidal choroidal vasculopathy"[tiab] OR "pachychoroid neovasculopathy"[tiab] OR "pachychoroid"[tiab] OR "PCV"[tiab]	**76,793**
**#3**	**#1 AND #2**	**97**

	**Web of Science**	**Total**
**#1**	TS=("aflibercept 8 mg" OR "aflibercept 8mg" OR "aflibercept 8-mg" OR "high-dose aflibercept" OR "high dose aflibercept" OR "Eylea HD")	**179**
**#2**	TS=("macular degeneration" OR "neovascular age-related macular degeneration" OR "neovascular age related macular degeneration" OR "neovascular AMD" OR "nAMD" OR "wAMD" OR "AMD" OR "ARMD" OR "wet AMD" OR "wet age-related macular degeneration" OR "wet age related macular degeneration" OR "wet macular degeneration" OR "exudative AMD" OR "exudative age-related macular degeneration" OR "exudative macular degeneration" OR "choroidal neovascularization" OR "choroidal neovascularisation" OR "macular neovascularization" OR "macular neovascularisation" OR "CNV" OR "CNVM" OR "MNV" OR "age-related maculopath*" OR "age related maculopath*" OR "retinal angiomatous proliferation" OR "polypoidal choroidal vasculopathy" OR "pachychoroid neovasculopathy" OR "pachychoroid" OR "PCV")	**83,648**
**#3**	**#1 AND #2**	**119**

	**Cochrane Central Register of Controlled Trials (CENTRAL)**	**Total**
	Title Abstract Keyword was used	
**#1**	("aflibercept 8 mg" OR "aflibercept 8mg" OR "aflibercept 8-mg" OR "high-dose aflibercept" OR "high dose aflibercept" OR "Eylea HD")	**62**
**#2**	("macular degeneration" OR "neovascular age-related macular degeneration" OR "neovascular age related macular degeneration" OR "neovascular AMD" OR "nAMD" OR "wAMD" OR "AMD" OR "ARMD" OR "wet AMD" OR "wet age-related macular degeneration" OR "wet age related macular degeneration" OR "wet macular degeneration" OR "exudative AMD" OR "exudative age-related macular degeneration" OR "exudative macular degeneration" OR "choroidal neovascularization" OR "choroidal neovascularisation" OR "macular neovascularization" OR "macular neovascularisation" OR "CNV" OR "CNVM" OR "MNV" OR (age-related NEXT maculopath*) OR (age NEXT related NEXT maculopath*) OR "retinal angiomatous proliferation" OR "polypoidal choroidal vasculopathy" OR "pachychoroid neovasculopathy" OR "pachychoroid" OR "PCV")	**7,089**
**#3**	**#1 AND #2**	**42**

	**Scopus**	**Total**
**#1**	TITLE-ABS-KEY("aflibercept 8 mg" OR "aflibercept 8mg" OR "aflibercept 8-mg" OR "high-dose aflibercept" OR "high dose aflibercept" OR "Eylea HD")	**118**
**#2**	TITLE-ABS-KEY("macular degeneration" OR "neovascular age-related macular degeneration" OR "neovascular age related macular degeneration" OR "neovascular AMD" OR "nAMD" OR "wAMD" OR "AMD" OR "ARMD" OR "wet AMD" OR "wet age-related macular degeneration" OR "wet age related macular degeneration" OR "wet macular degeneration" OR "exudative AMD" OR "exudative age-related macular degeneration" OR "exudative macular degeneration" OR "choroidal neovascularization" OR "choroidal neovascularisation" OR "macular neovascularization" OR "macular neovascularisation" OR "CNV" OR "CNVM" OR "MNV" OR "age-related maculopath*" OR "age related maculopath*" OR "retinal angiomatous proliferation" OR "polypoidal choroidal vasculopathy" OR "pachychoroid neovasculopathy" OR "pachychoroid" OR "PCV")	105,140
**#3**	**#1 AND #2**	**93**

	Embase	Total
#1	'aflibercept 8 mg':ti,ab,kw OR 'aflibercept 8mg':ti,ab,kw OR 'aflibercept 8-mg':ti,ab,kw OR 'high-dose aflibercept':ti,ab,kw OR 'high dose aflibercept':ti,ab,kw OR 'eylea hd':ti,ab,kw	214
#2	'macular degeneration'/exp OR 'retina macula lutea degeneration' OR 'choroidal neovascularization'/exp	64,869
#3	'macular degeneration':ti,ab,kw OR 'neovascular age-related macular degeneration':ti,ab,kw OR 'neovascular age related macular degeneration':ti,ab,kw OR 'neovascular amd':ti,ab,kw OR 'namd':ti,ab,kw OR 'wamd':ti,ab,kw OR 'amd':ti,ab,kw OR 'armd':ti,ab,kw OR 'wet amd':ti,ab,kw OR 'wet age-related macular degeneration':ti,ab,kw OR 'wet age related macular degeneration':ti,ab,kw OR 'wet macular degeneration':ti,ab,kw OR 'exudative amd':ti,ab,kw OR 'exudative age-related macular degeneration':ti,ab,kw OR 'exudative macular degeneration':ti,ab,kw OR 'choroidal neovascularization':ti,ab,kw OR 'choroidal neovascularisation':ti,ab,kw OR 'macular neovascularization':ti,ab,kw OR 'macular neovascularisation':ti,ab,kw OR 'cnv':ti,ab,kw OR 'cnvm':ti,ab,kw OR 'mnv':ti,ab,kw OR 'age-related maculopath*':ti,ab,kw OR 'age related maculopath*':ti,ab,kw OR 'retinal angiomatous proliferation':ti,ab,kw OR 'polypoidal choroidal vasculopathy':ti,ab,kw OR 'pachychoroid neovasculopathy':ti,ab,kw OR 'pachychoroid':ti,ab,kw OR 'pcv':ti,ab,kw	91,564
#4	#2 OR #3	109,931
#5	**#1 AND #4**	169

Clinical trial.gov 12

"aflibercept 8 mg" AND "macular degeneration"

Google Scholar first 200 by relevance

"aflibercept 8 mg" AND macular degeneration

- A total of 732 were uploaded to EndNote X9. 411 unique records after deduplication.

Supplementary Table S1. GRADE Certainty of Evidence Assessment for Co-Primary Outcomes

Outcome	k	Total eyes	Effect estimate [95% CI]	Risk of bias	Inconsistency	Indirectness	Imprecision	Publication bias	Overall certainty	Explanation
**BCVA change (logMAR)**	18	1,274	−0.017 logMAR [−0.027, −0.007]	Not serious	Not serious	Not serious	Not serious	Not serious	⨁⨁○○ Low	Observational evidence (starts low). No domains downgraded. $I^2 = 0.0\%$; Egger $p = 0.771$; CI excludes clinically meaningful harm.
**CST change (µm)**	18	1,365	−21.5 µm [−29.3, −13.7]	Not serious	Serious ↓	Not serious	Not serious	Not serious	⨁○○○ Very low	Observational evidence (starts low). Downgraded one level for inconsistency: $I^2 = 56.0\%$ (moderate); no prespecified moderator substantially reduced residual heterogeneity (range: 54.6–58.4%). Egger $p = 0.048$ noted but not downgraded (small-study effects, not selective reporting). Trim-and-fill analysis confirmed robustness (adjusted WMD: −17.8 µm, $p < 0.001$ after imputing 4 studies).
**Interval change (wk)**	10	908	+1.79 wk [+1.32, +2.27]	Not serious	Serious ↓	Not serious	Not serious	Not serious	⨁○○○ Very low	Observational evidence (starts low). Downgraded one level for inconsistency: $I^2 = 74.3\%$ (substantial). Reason for switch partially explained heterogeneity (residual $I^2 = 54.5\%$) but substantial unexplained variability remained. Egger $p = 0.783$. One level downgrade applied rather than two because the reason-for-switch moderator partially explained heterogeneity, and the direction of effect was consistent across all 10 studies.

All included studies are observational (single-arm, pre–post design); evidence begins at low certainty per GRADE guidance for observational studies.
↓ indicates downgrade applied for this domain.

Supplementary Table S2. Reasons for Exclusions at Full-Text Screen Phase.

Study ID	Study Name	Reason for Exclusion
**Airody 2025**	SPECTRUM: EARLY RESULTS FROM THE FIRST GLOBAL REALWORLD STUDY OF AFLIBERCEPT 8 MG IN PATIENTS WITH PRETREATED DIABETIC MACULAR OEDEMA	Non-nAMD indication (DMO cohort of SPECTRUM trial, NCT06075147).
**Anakwenze 2025**	From: Real World Experience of Aflibercept 8mg at York and Scarborough Teaching Hospitals NHS Foundation trust for neovascular Age related Macula Degeneration (nAMD) and Diabetic Macular Edema (DME).	Abstract only.
**Arnold 2026**	Early Real-World Outcomes of Intravitreal Aflibercept 8 Mg in Neovascular Age-Related Macular Degeneration: A Multinational Retrospective Study	Abstract only.
**Bailey 2025**	SPECTRUM: EARLY RESULTS FROM THE FIRST GLOBAL REALWORLD STUDY OF AFLIBERCEPT 8MG IN PATIENTS WITH PRETREATED NEOVASCULAR AGE-RELATED MACULAR DEGENERATION	Abstract only (early enrollment update for SPECTRUM previously treated nAMD cohort; reports cumulative enrollment status, not patient-level outcomes).
**Bartolomeo 2026**	Early Outcomes of Intravitreal Aflibercept 8 mg in Eyes Previously Treated with Aflibercept 2 mg for Neovascular Age-Related Macular Degeneration with AI-Based Biomarker Quantification	Duplicate publication. Bartolomeo's single-center cohort at Swiss Visio Montchoisi, Lausanne (n=52, 6-month follow-up) is incorporated into the larger Swiss Retina Research Network multicenter analysis by Kitay 2026, since Swiss Visio Montchoisi is one of the 11 participating SRRN centers. Kitay is retained as the more complete report (larger sample of 283 eyes, 12-month follow-up, multicenter), and Bartolomeo is excluded as a duplicate report of the same patient cohort.
**Broadhead 2026**	Early Real-World Efficacy and Safety of Aflibercept 8 Mg in Australian Patients with Neovascular Age- Related Macular Degeneration	Abstract only.
**Chang 2025**	Faricimab versus Aflibercept 8mg in the Treatment of Eyes Recalcitrant to Alternative Anti-VEGF Therapies	Abstract only.

**Donia 2025**	Real-world efficacy, safety, and durability of aflibercept 8 mg in neovascular age-related macular degeneration	Abstract only.
	EarLy Treatment Response in nEoVascular Macular Degeneration With Eylea 8mg: ELEV8	Trial registration only, no results posted.
**Ertan 2020**	Switch to aflibercept in the treatment of neovascular age-related macular degeneration: 30-month results	Wrong intervention. Investigates patients switching from ranibizumab to aflibercept 2 mg, not aflibercept 8 mg.
**Fein 2025**	Clinical Outcomes in Neovascular Age-related Macular Degeneration with Aflibercept 8 mg in the Phase II CANDELA Study	Treatment-naïve only, no switch cohort. Post hoc analysis of the Phase 2 CANDELA trial, which enrolled exclusively treatment-naïve nAMD patients.
**Gartaganis 2025**	The Effect of Using High Dose Aflibercept 8mg in Treating Age-Related Macular Degeneration	Abstract only.
**Gondal 2025**	Efficacy and safety of three-monthly loading doses of intravitreal injections of Aflibercept 8 mg in clinical practice at a district general hospital in the United Kingdom	Abstract only.
	High Dose (HD) Aflibercept Switch in Neovascular Age-related Macular Degeneration (nAMD): to Load or Maintain (HEIRLOOM)	Trial registration only, no results posted.
	Home Monitoring in eAMD Treatment 2026	Trial registration only, no results posted.
**Leng 2025**	Early insights from real-world use of aflibercept 8 mg among eyes with neovascular age-related macular degeneration (nAMD) switching from other anti-VEGF agents	Abstract only.

**Liesenhoff 2025**	High-Dose 8 mg Aflibercept for Neovascular Age-Related Macular Degeneration: Who Is Being Treated with This New Agent?	No co-primary outcome reported. Describes the indication spectrum for switching to aflibercept 8 mg in clinical practice but does not report changes in BCVA, CST, or treatment interval beyond baseline.
**Liesenhoff 2026**	Efficacy of 2 mg vs. High-Dose 8 mg Aflibercept in Neovascular Age Related Macular Degeneration	Inaccessible full text.
**Matsumoto 2024**	Retinal vasculitis after intravitreal aflibercept 8 mg for neovascular age-related macular degeneration	Mixed cohort, switch subgroup not separately reported. Combined cohort of 18 treatment-naïve and 17 switch eyes; outcomes not separately reported for the previously treated subgroup.
**Menasalvas 2026**	4CPS-128 Aflibercept schemes in a third-level hospital: real-word experience and cost analysis	Abstract only.
**Mojumder 2025**	Clinical Experience with Aflibercept 8 mg in High Need Treatment-Experienced Patients with Neovascular Age-Related Macular Degeneration	Abstract only.
	An Observational Study Program to Investigate the Effectiveness of Aflibercept 8 mg Used in DME and nAMD in a Real-world Setting	Trial registration only, no results posted.
	A Phase 3b Single-Arm Study of Aflibercept 8 mg in Participants With Neovascular Age-Related Macular Degeneration (nAMD) or Diabetic Macular Edema (DME)	Trial registration only, no results posted.
	Real-World Patient Characteristics, Treatment Patterns, and Outcomes Among Patients With Neovascular Age-Related Macular Degeneration (nAMD) and Diabetic Macular Edema (DME) Treated With Aflibercept 8 mg in the United States	Trial withdrawn.
	Retrospective Multicenter Real-world Observational Study of Switching to Aflibercept 8 mg in Patients With Refractory or Dependent Exudative Age-related Macular Degeneration	Trial registration only, no results posted.

**Sanchez 2026**	4CPS-193 Descriptive real-world outcomes with aflibercept 8 mg/0.07 ml in wet age-related macular degeneration and diabetic macular oedema	Abstract only.
**Sather 2026**	Real-World Durability of Aflibercept 8 mg and Faricimab in Initiators Versus Switchers: A Multicenter Retrospective Analysis	Non-nAMD indication without separable nAMD data. Switch cohort (Cohort 3, n=223 eyes) is composed of mixed indications (nAMD, DME, RVO); raw outcomes reported only for the combined group, and adjusted regression coefficients in Tables 3 to 5 do not provide the separable raw nAMD-only data required for meta-analytic synthesis.
**Stoehr 2025**	Intraocular inflammation following aflibercept 8 mg: real-world data from a multicentre retrospective observational study	Fewer than 10 eyes of eligible indication. Series of 12 eyes with intraocular inflammation events, of which only 9 have the eligible nAMD indication (2 had DME). Fails the protocol section 4.5 threshold of at least 10 eyes per study. Additionally a safety case series of adverse events rather than a switch cohort with paired pre/post co-primary outcomes.
	Efficacy of Switching to Aflibercept 8mg in Patients with Neovascular AMD Showing Limited Response to Faricimab or Aflibercept 2mg	Trial registration only, no results posted.
**Tamai 2025**	Switching From Aflibercept 2 mg to 8 mg in Vitrectomized Eyes With Neovascular Age-Related Macular Degeneration	Fewer than 10 eyes of eligible nAMD indication.

Supplementary Table S3. Comparison of GLMM-Logit and Freeman-Tukey Pooled Estimates for Proportion Outcomes

Outcome	GLMM-logit [95% CI]	Freeman-Tukey [95% CI]
IRF resolution	37.5% [27.7, 48.5]	37.5% [26.1, 49.6]
SRF resolution	37.5% [29.7, 46.1]	37.6% [29.5, 46.1]
≥8-week achievement	69.8% [43.2, 87.6]	65.7% [45.0, 83.7]
≥12-week achievement	13.7% [5.9, 28.8]	13.7% [5.0, 25.3]
≥16-week achievement	9.6% [3.6, 23.1]	11.0% [3.1, 22.6]
IOI incidence	0.2% [0.0, 1.0]	0.1% [0.0, 0.6]
Discontinuation	19.9% [13.3, 28.7]	19.9% [12.3, 28.7]

GLMM-logit, generalized linear mixed model with logit link (primary method); Freeman-Tukey, double-arcsine transformation with DerSimonian–Laird estimation and back-transformation using the harmonic mean of sample sizes (sensitivity method). Both methods yielded consistent estimates across all proportion outcomes.

Supplementary Table S4. Sensitivity Analyses for Co-Primary Outcomes

Analysis	BCVA (logMAR)		CST (μm)		Interval (wk)	
	WMD [95% CI]	I^2	WMD [95% CI]	I^2	WMD [95% CI]	I^2
**Primary**	−0.017 [−0.027, −0.007]	0.00%	−21.5 [−29.3, −13.7]	56.00%	+1.79 [+1.32, +2.27]	74.30%
**Excl. <20 eyes**	−0.016 [−0.027, −0.005]	0.00%	−19.1 [−24.9, −13.2]	40.80%	+1.79 [+1.32, +2.27]	74.30%
**Excl. FU <3 mo**	−0.019 [−0.031, −0.007]	0.00%	−18.2 [−24.6, −11.8]	42.00%	N/A	—
**Excl. single-inj**	−0.017 [−0.028, −0.005]	0.00%	−19.4 [−26.1, −12.7]	46.90%	N/A	—
**Excl. pure PRN**	N/A	—	N/A	—	N/A	—
**Excl. label jurisd.**	−0.015 [−0.028, −0.002]	4.30%	−21.1 [−28.2, −14.0]	39.70%	+1.66 [+0.74, +2.58]	83.70%
**ETDRS-only**	+0.002 [−0.014, +0.018]	0.00%	N/A	—	N/A	—
**r = 0.3**	−0.017 [−0.027, −0.007]	0.00%	−21.0 [−28.3, −13.7]	46.30%	+1.81 [+1.35, +2.28]	68.20%
**r = 0.7**	−0.015 [−0.026, −0.005]	5.00%	−22.2 [−30.5, −13.9]	67.60%	+1.76 [+1.27, +2.26]	81.40%
**Egger/Peters p**	0.771		0.048		0.783	

WMD, weighted mean difference; CI, confidence interval; I^2, percentage of variability due to heterogeneity. Correlation sensitivity refers to the assumed pre–post correlation coefficient (r) used for imputing the standard deviation of the within-subject change when not reported. Label-constraint jurisdictions are the United States and Japan, where regulatory labelling mandates minimum injection intervals of ≥8 weeks after the loading phase. ETDRS-only restricts to studies that natively reported visual acuity in ETDRS letters (excluding those requiring logMAR-to-ETDRS conversion). N/A, not applicable to this outcome.

Supplementary Table S5. Subgroup Analyses for BCVA (logMAR)

Subgroup	k	WMD [95% CI]	p	Test of moderators
**Region**				**F(4,14) = 3.18, p = 0.047**
Asia	6	−0.020 [−0.042, +0.002]	0.072	
Europe	8	−0.018 [−0.032, −0.005]	0.012	
North America	3	−0.015 [−0.058, +0.029]	0.482	
Other	1	0.000 [−0.034, +0.034]	1.000	
**Follow-up duration**				**F(2,16) = 6.10, p = 0.011**
<6 months	5	−0.015 [−0.028, −0.002]	0.023	
≥6 months	13	−0.019 [−0.035, −0.002]	0.028	
**Reason for switch**				**F(4,14) = 5.16, p = 0.009**
Extension goal	3	−0.027 [−0.041, −0.012]	0.001	
Mixed/unspecified	4	−0.002 [−0.019, +0.015]	0.796	
Persistent disease	6	−0.017 [−0.037, +0.003]	0.083	
Refractory disease	5	−0.018 [−0.053, +0.017]	0.296	
**Loading phase after switch**				**F(2,16) = 6.07, p = 0.011**
No loading	15	−0.016 [−0.027, −0.006]	0.005	
Loading administered	3	−0.021 [−0.054, +0.012]	0.199	
**Regulatory label constraint**				**F(2,16) = 6.02, p = 0.011**
No constraint	14	−0.016 [−0.028, −0.005]	0.009	
Constraint (US/Japan)	4	−0.018 [−0.039, +0.003]	0.091	
**Prior aflibercept 2 mg majority**				**F(2,16) = 6.02, p = 0.011**
No	4	−0.020 [−0.056, +0.017]	0.276	
Yes	14	−0.016 [−0.027, −0.006]	0.005	

WMD, weighted mean difference; CI, confidence interval. Subgroup estimates are from mixed-effects meta-regression (DerSimonian–Laird + HKSJ) with the moderator entered as a categorical variable without intercept. Test of moderators assesses whether the pooled effect differs across subgroup levels. Label-constraint jurisdictions: United States and Japan.

Supplementary Table S6. Subgroup Analyses for CST (µm)

Subgroup	k	WMD [95% CI]	p	Test of moderators	Residual I²
**Region**				**F(4,14) = 8.17, p = 0.001**	**54.6%**
Asia	4	−25.1 [−40.9, −9.3]	0.004		
Europe	8	−19.8 [−31.6, −7.9]	0.003		
North America	3	−16.1 [−36.6, +4.5]	0.117		
Other	1	−39.0 [−74.6, −3.4]	0.034		
**Follow-up duration**				**F(2,16) = 15.85, p < 0.001**	**55.6%**
<6 months	5	−22.7 [−36.2, −9.1]	0.003		
≥6 months	13	−21.0 [−31.2, −10.9]	<0.001		
**Reason for switch**				**F(4,14) = 7.68, p = 0.002**	
Extension goal	3	−18.8 [−37.3, −0.4]	0.046		
Mixed/unspecified	4	−28.2 [−44.1, −12.3]	0.002		
Persistent disease	6	−18.0 [−32.5, −3.5]	0.018		
Refractory disease	5	−23.1 [−47.0, +0.8]	0.058		
**Loading phase after switch**				**F(2,16) = 17.10, p < 0.001**	**56.7%**
No loading	15	−20.3 [−28.7, −11.8]	<0.001		
Loading administered	3	−30.5 [−52.9, −8.0]	0.011		
**Regulatory label constraint**				**F(2,16) = 15.76, p < 0.001**	**56.6%**
No constraint	14	−21.8 [−31.0, −12.6]	<0.001		
Constraint (US/Japan)	4	−21.1 [−38.6, −3.5]	0.022		
**Prior aflibercept 2 mg majority**				**F(2,16) = 16.24, p < 0.001**	**58.4%**
No	4	−16.8 [−37.0, +3.4]	0.097		
Yes	14	−22.5 [−31.3, −13.7]	<0.001		

CST, central subfield thickness. See Supplementary Table S5 footnote for methodological details.

Supplementary Table S7. Subgroup Analyses for Treatment Interval (weeks)

Subgroup	k	WMD [95% CI]	p	Test of moderators	Residual I²
**Region**				**F(3,7) = 18.33, p = 0.001**	**78.3%**
Asia	4	+1.67 [+0.71, +2.63]	0.005		
Europe	5	+1.88 [+1.10, +2.66]	<0.001		
North America	1	+1.60 [−0.01, +3.21]	0.051		
**Follow-up duration**				**F(2,8) = 62.81, p < 0.001**	**59.1%**
<6 months	4	+2.35 [+1.70, +3.00]	<0.001		
≥6 months	6	+1.53 [+1.06, +2.00]	<0.001		
**Reason for switch**				**F(4,6) = 37.62, p < 0.001**	**54.5%**
Extension goal	3	+2.22 [+1.52, +2.92]	<0.001		
Mixed/unspecified	2	+1.90 [+1.00, +2.79]	0.002		
Persistent disease	3	+1.16 [+0.51, +1.81]	0.005		
Refractory disease	2	+2.23 [+1.41, +3.04]	<0.001		
**Loading phase after switch**				**F(2,8) = 34.89, p < 0.001**	**75.0%**
No loading	7	+1.68 [+1.10, +2.26]	<0.001		
Loading administered	3	+2.07 [+1.12, +3.03]	0.001		
**Regulatory label constraint**				**F(2,8) = 32.37, p < 0.001**	**77.1%**
No constraint	6	+1.69 [+1.03, +2.35]	<0.001		
Constraint (US/Japan)	4	+1.92 [+1.11, +2.73]	<0.001		

See Supplementary Table S5 footnote for methodological details. North America contains only one study (Bala 2025); the subgroup estimate for this stratum should be interpreted with caution.

Supplementary Table S8. Correlation Sensitivity Analysis for PED Height Change

Correlation (r)	WMD [95% CI] (μm)	I^2
r = 0.3	−22.9 [−31.3, −14.4]	34.8%
r = 0.5 (primary)	−22.9 [−31.2, −14.6]	35.4%
r = 0.7	−23.1 [−31.0, −15.1]	36.8%
r = 0.8	−23.2 [−30.8, −15.5]	38.2%

WMD, weighted mean difference; CI, confidence interval; I^2, percentage of variability due to heterogeneity. The primary analysis assumes r = 0.5; additional sensitivity analyses at r = 0.3, 0.7, and 0.8 are shown. The r = 0.8 sensitivity was prespecified for PED given the high temporal stability of PED measurements.

Studies Excluded from Quantitative Pooling of Co-Primary Outcomes

BCVA change: 3 studies excluded (18 of 21 pooled)

Angus 2026 was excluded from the BCVA meta-analysis because mean baseline and final visual acuity values were reported without any accompanying measure of dispersion. In the absence of standard deviations, confidence intervals, interquartile ranges, or standard errors, the variance of the effect estimate could not be calculated or imputed.

Bates 2025 was excluded for the same reason. Although mean logMAR values were reported at baseline and at each subsequent injection visit, no measure of dispersion was provided for any timepoint. The large sample size of the previously treated nAMD subgroup made imputation from external sources inappropriate given the potential to disproportionately influence the pooled estimate.

Mizuma 2026 was excluded because BCVA outcomes were reported only for the combined cohort of 40 eyes, including both switch and treatment-naïve eyes, with no separate reporting for the previously treated subgroup. The corresponding author was contacted to request switch-subgroup-specific data; a reply was pending at the time of analysis.

CST change: 3 studies excluded (18 of 21 pooled)

Bates 2025 was excluded because the study explicitly noted that imaging data were not captured in the registry database. Central subfield thickness was therefore not reported for any subgroup, and no anatomical data were available for extraction.

Angus 2026 was excluded because mean CST values were reported at baseline and final follow-up without any accompanying measure of dispersion. The study-level variance could not be estimated and the effect size could not be incorporated into the random-effects model.

Mizuma 2026 was excluded for the same reason as for BCVA. Central subfield thickness was reported only for the combined cohort, and switch-subgroup-specific data were not available. The corresponding author was contacted; a reply was pending at the time of analysis.

Treatment interval change : 11 studies excluded (10 of 21 pooled)

Hara 2025 and Mizukami 2025 were excluded because both studies assessed outcomes after a single injection of aflibercept 8 mg. Treatment interval extension cannot be evaluated after a single dose, as the post-switch interval reflects the pre-existing schedule rather than a clinician-directed adjustment based on drug response. This exclusion was prespecified in the study protocol.

Bailey 2026 and Chi 2025 were excluded because treatment interval data were not reported in the published manuscripts.

Grün 2025 was excluded because treatment interval outcomes were reported only for the combined cohort of pretreated and treatment-naïve eyes, without separate data for the previously treated subgroup. The corresponding author was contacted to request subgroup-specific interval data; a reply was pending at the time of analysis.

Angus 2026, Bates 2025, Hafner 2025, Momenaei 2025, Palm 2025, and Sambhara 2025 were excluded because mean pre-switch and post-switch treatment intervals were reported without any accompanying measure of dispersion. Without standard deviations, interquartile ranges, or confidence intervals, the variance of the within-subject interval change could not be calculated or imputed.

	Q1 Inclusion clear?	Q2 Condition reliable?	Q3 Valid identification?	Q4 Consecutive?	Q5 Complete inclusion?	Q6 Demographics?	Q7 Clinical info?	Q8 Outcomes/ follow–up?	Q9 Sites/clinics demo?	Q10 Stats appropriate?	Overall
Abu Ishkheidem 2025	Y	Y	Y	U	Y	Y	Y	Y	Y	Y	Low
Angus 2026	Y	Y	Y	Y	U	Y	Y	Y	Y	N	Low
Bailey 2026	Y	Y	Y	U	U	Y	Y	Y	Y	Y	Low
Bala 2025	Y	Y	Y	Y	Y	Y	Y	Y	Y	Y	Low
Bates 2025	Y	Y	Y	U	U	Y	Y	Y	Y	Y	Low
Cheung 2026	Y	Y	Y	Y	U	Y	Y	Y	Y	Y	Low
Chi 2025	Y	Y	Y	U	U	Y	Y	Y	Y	Y	Low
Emfietzoglou 2026	Y	Y	Y	Y	U	Y	Y	Y	Y	Y	Low
Grün 2025	Y	Y	Y	U	Y	Y	Y	Y	Y	Y	Low
Hafner 2025	Y	Y	Y	U	U	Y	Y	Y	Y	Y	Low
Hara 2025	Y	Y	Y	Y	Y	Y	Y	Y	Y	Y	Low
Janmohamed 2026	Y	Y	Y	Y	U	Y	Y	Y	Y	Y	Low
Kataoka 2026	Y	Y	Y	Y	Y	Y	Y	Y	Y	Y	Low
Kindo 2026	Y	Y	Y	Y	U	Y	Y	Y	Y	Y	Low
Kitay 2026	Y	Y	Y	Y	Y	Y	Y	Y	Y	Y	Low
Mizukami 2025	Y	Y	Y	U	U	Y	Y	Y	Y	Y	Low
Mizuma 2026	Y	Y	Y	Y	Y	Y	Y	Y	Y	Y	Low
Momenaei 2025	Y	Y	Y	U	U	Y	Y	Y	Y	Y	Low
Musadiq 2025	Y	Y	Y	U	U	Y	Y	Y	Y	Y	Low
Palm 2025	Y	Y	Y	U	U	Y	Y	Y	Y	Y	Low
Sambhara 2025	Y	Y	Y	U	U	Y	Y	Y	Y	N	Moderate

= Yes = No = Unclear

Figure S1. Risk of bias assessment of included studies according to the JBI Critical Appraisal Checklist for Case Series.

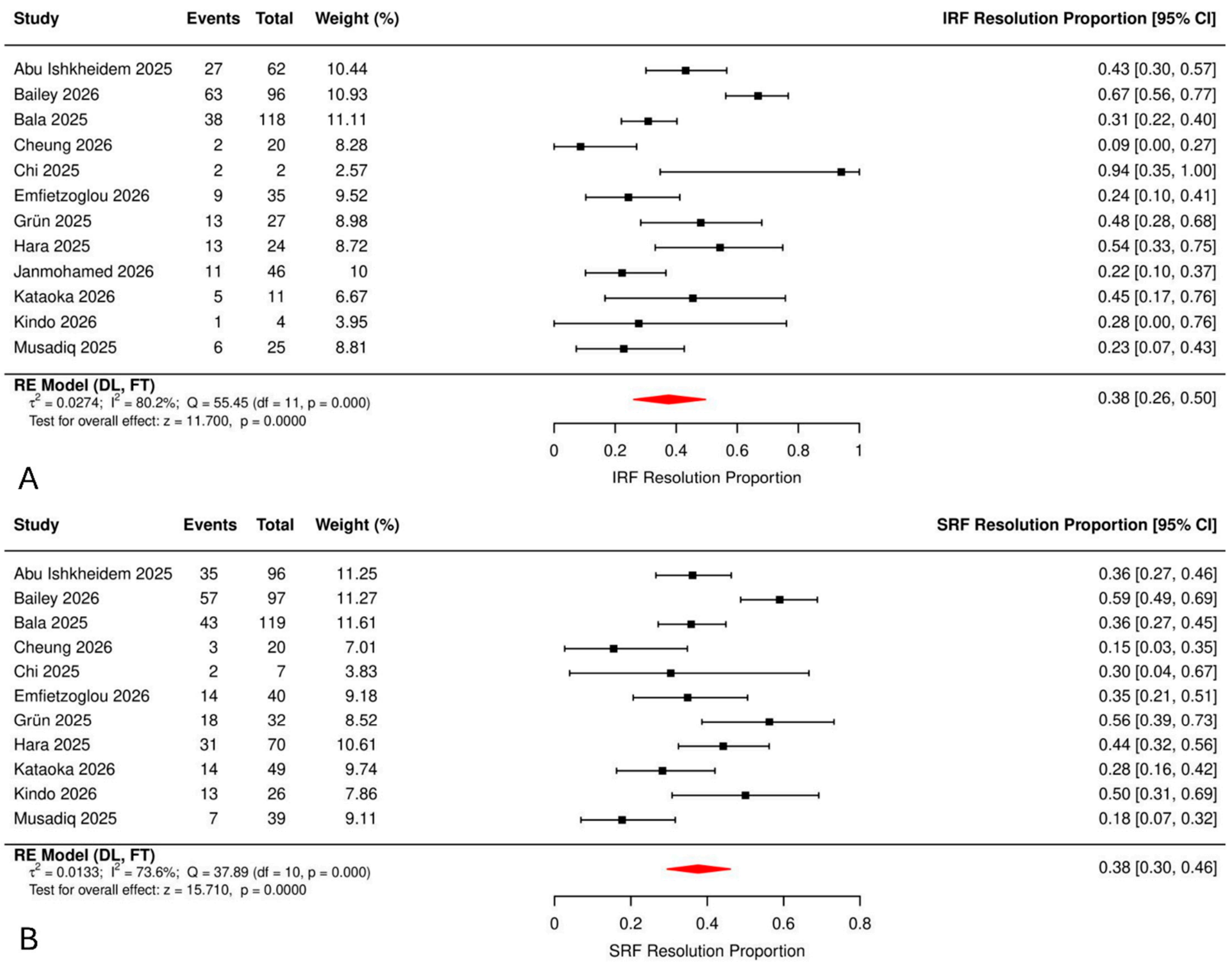

Figure S2. (A) Forest plot of pooled intraretinal fluid (IRF) (B) Forest plot of pooled subretinal fluid (SRF) resolution rates after switching to aflibercept 8 mg. The pooled proportions displayed reflect the Freeman-Tukey double-arcsine transformation; the primary GLMM-logit estimates are reported in the text.

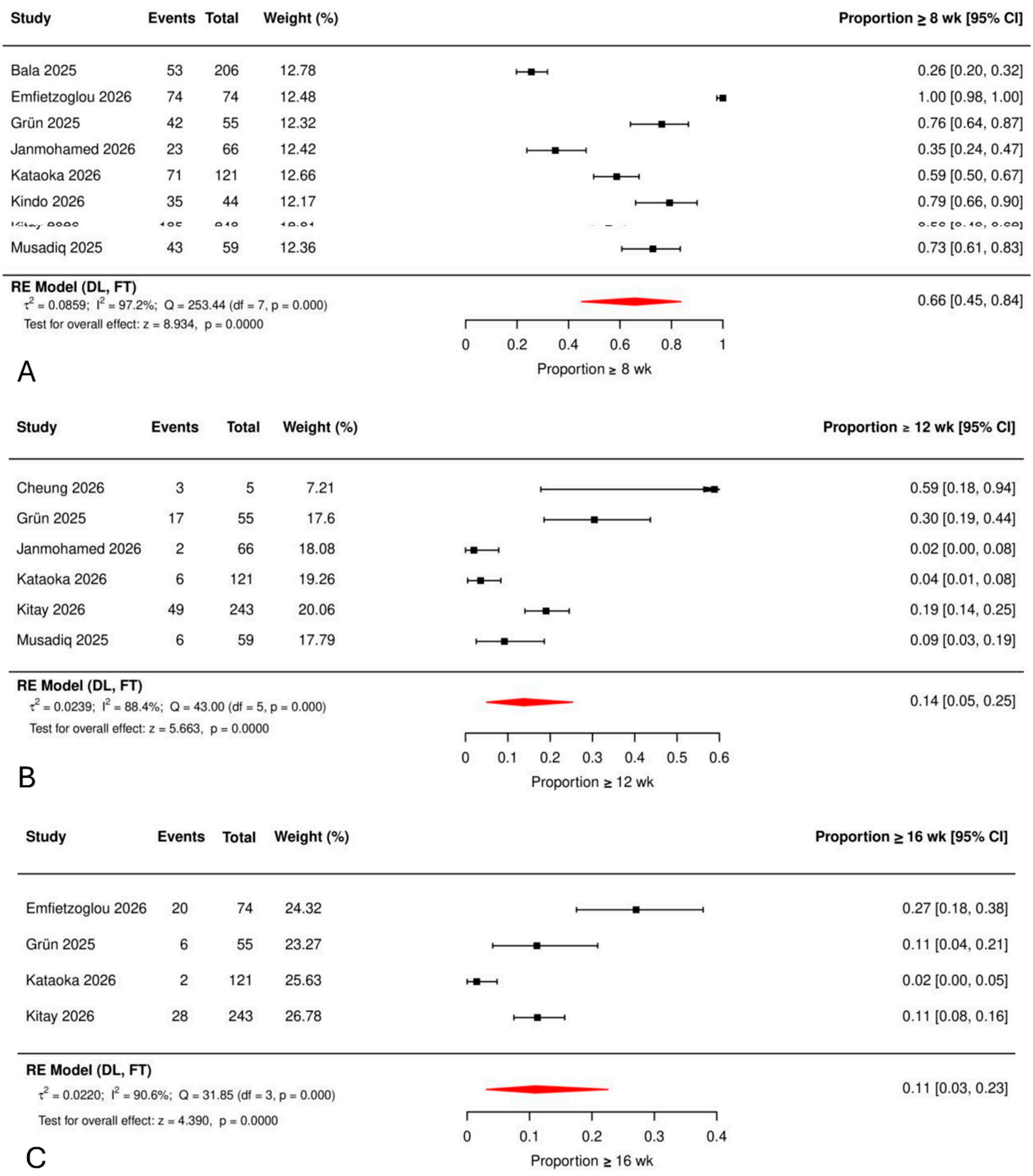

Figure S3. Forest plots of pooled proportions of eyes achieving treatment intervals of (A) ≥8 weeks, (B) ≥12 weeks, and (C) ≥16 weeks after switching to aflibercept 8 mg. Pooled proportions were estimated using the Freeman–Tukey double-arcsine transformation with DerSimonian–Laird estimation.

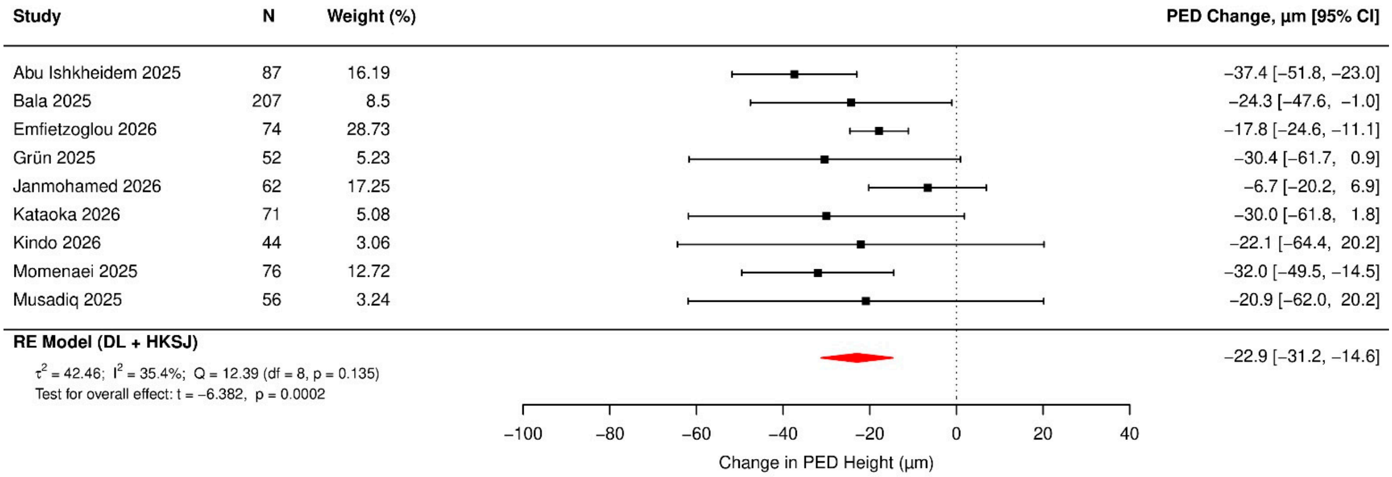

Figure S4. Forest plot of pooled change in pigment epithelial detachment (PED) height (µm) after switching to aflibercept 8 mg. The pooled weighted mean difference was estimated using a random-effects model (DerSimonian–Laird estimator with Hartung–Knapp–Sidik–Jonkman adjustment).

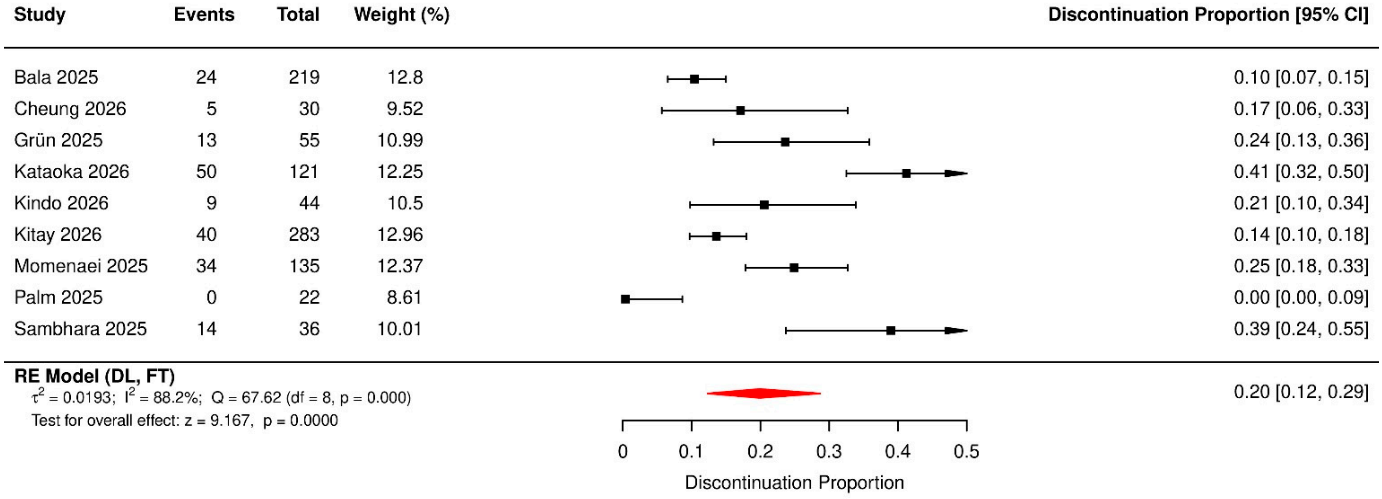

Figure S5. Forest plot of pooled discontinuation rate after switching to aflibercept 8 mg. The pooled proportion was estimated using the Freeman–Tukey double-arcsine transformation with DerSimonian–Laird estimation.

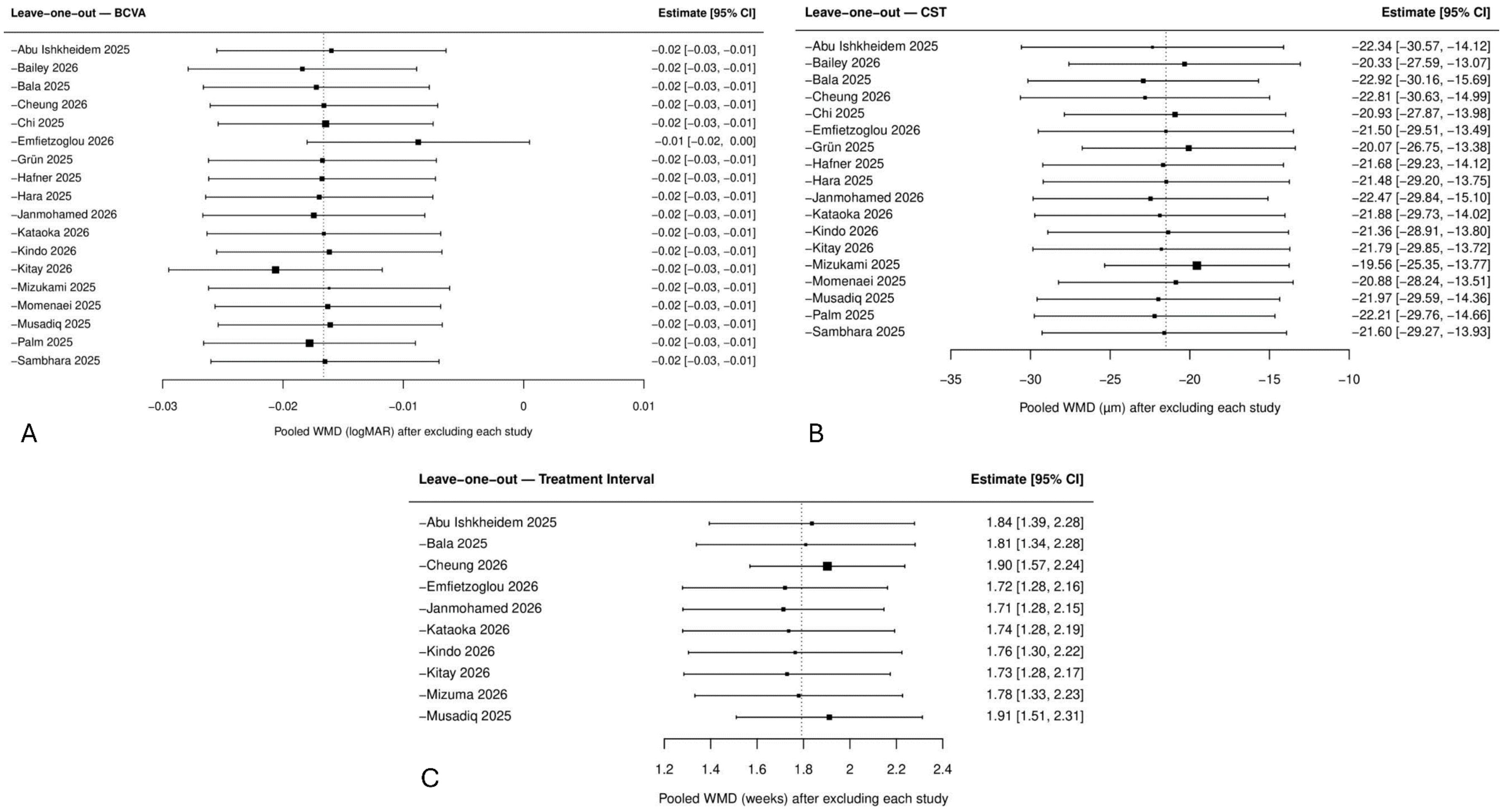

Figure S6. Leave-one-out sensitivity analyses for co-primary outcomes. Each row shows the pooled estimate after excluding the named study; vertical reference lines indicate the primary pooled estimates. (A) BCVA change, reference: −0.017 logMAR; (B) CST change, reference: −21.5 µm; (C) treatment interval change, reference: +1.79 weeks.

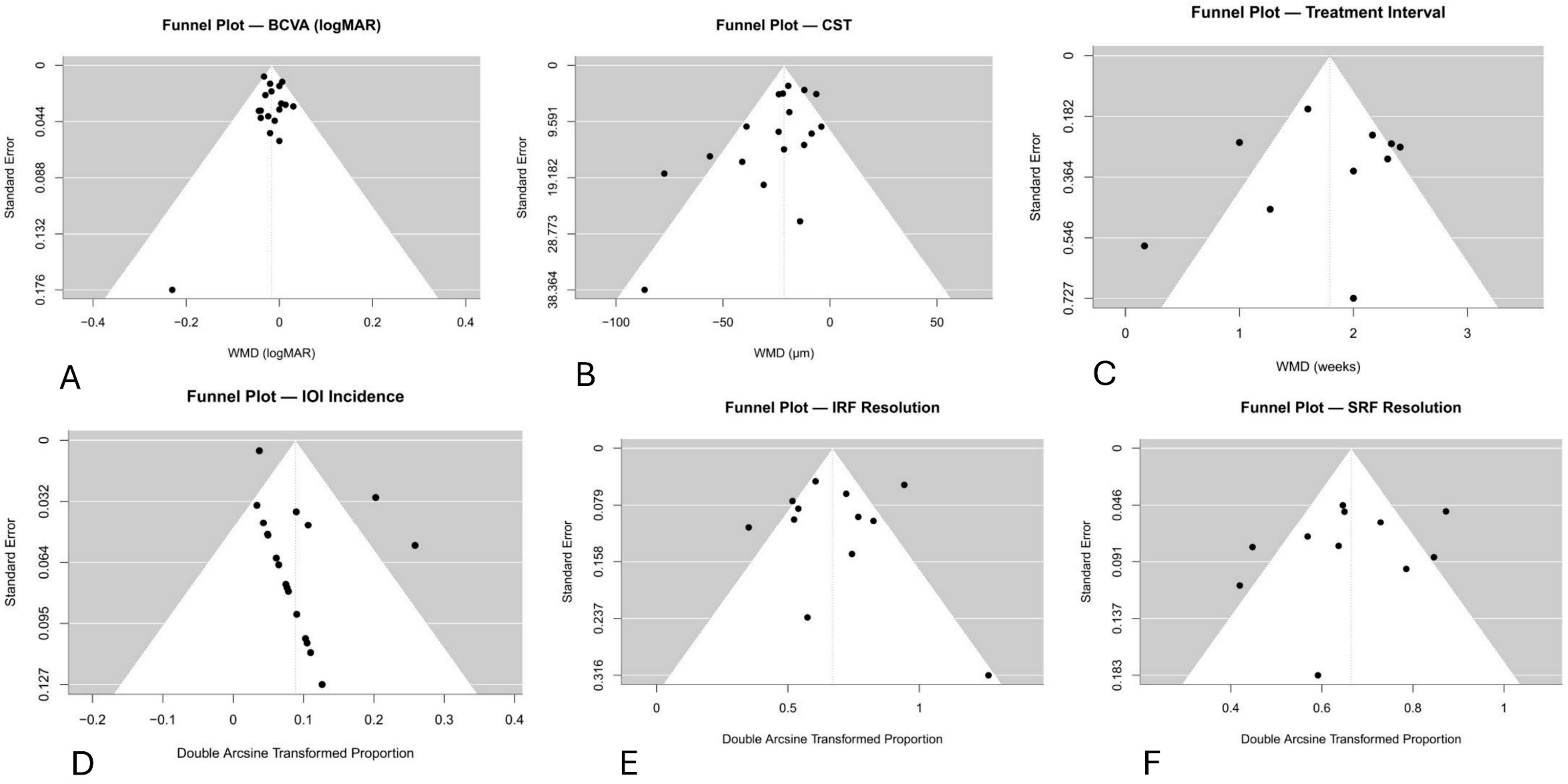

Figure S7. Funnel plots for publication bias assessment. (A) BCVA change, Egger p = 0.771; (B) CST change, Egger p = 0.048; (C) treatment interval change, Egger p = 0.783; (D) IOI incidence, Peters p = 0.002; (E) IRF resolution, Peters p = 0.473; (F) SRF resolution, Peters p = 0.4

PRISMA 2020 Checklist

Section and Topic	Item #	Checklist item	Location where item is reported
**TITLE**			
Title	1	Identify the report as a systematic review.	Title, p. 1
**ABSTRACT**			
Abstract	2	See the PRISMA 2020 for Abstracts checklist.	Abstract, p. 1
**INTRODUCTION**			
Rationale	3	Describe the rationale for the review in the context of existing knowledge.	Introduction, paragraphs 1–3
Objectives	4	Provide an explicit statement of the objective(s) or question(s) the review addresses.	Introduction, paragraph 4
**METHODS**			
Eligibility criteria	5	Specify the inclusion and exclusion criteria for the review and how studies were grouped for the syntheses.	Section 2.3
Information sources	6	Specify all databases, registers, websites, organisations, reference lists and other sources searched or consulted to identify studies. Specify the date when each source was last searched or consulted.	Section 2.2
Search strategy	7	Present the full search strategies for all databases, registers and websites, including any filters and limits used.	Supplementary Material, Search Strings, pp. 3–5
Selection process	8	Specify the methods used to decide whether a study met the inclusion criteria of the review, including how many reviewers screened each record and each report retrieved, whether they worked independently, and if applicable, details of automation tools used in the process.	Section 2.3
Data collection process	9	Specify the methods used to collect data from reports, including how many reviewers collected data from each report, whether they worked independently, any processes for obtaining or confirming data from study investigators, and if applicable, details of automation tools used in the process.	Section 2.4
Data items	10a	List and define all outcomes for which data were sought. Specify whether all results that were compatible with each outcome domain in each study were sought (e.g. for all measures, time points, analyses), and if not, the methods used to decide which results to collect.	Section 1, paragraph 4; Section 2.4
	10b	List and define all other variables for which data were sought (e.g. participant and intervention characteristics, funding sources). Describe any assumptions made about any missing or unclear information.	Section 2.4
Study risk of bias assessment	11	Specify the methods used to assess risk of bias in the included studies, including details of the tool(s) used, how many reviewers assessed each study and whether they worked independently, and if applicable, details of automation tools used in the process.	Section 2.5
Effect measures	12	Specify for each outcome the effect measure(s) (e.g. risk ratio, mean difference) used in the synthesis or presentation of results.	Section 2.6
Synthesis methods	13a	Describe the processes used to decide which studies were eligible for each synthesis (e.g. tabulating the study intervention characteristics and comparing against the planned groups for each synthesis (item #5)).	Supplementary Material, Studies Excluded from Quantitative Pooling, p. 16
	13b	Describe any methods required to prepare the data for presentation or synthesis, such as handling of missing summary statistics, or data conversions.	Section 2.4
	13c	Describe any methods used to tabulate or visually display results of individual studies and syntheses.	Figures 2–5; Supplementary Figures S2–S7
	13d	Describe any methods used to synthesize results and provide a rationale for the choice(s). If meta-analysis was performed, describe the model(s), method(s) to identify the presence and extent of statistical heterogeneity, and software package(s) used.	Section 2.6

PRISMA 2020 Checklist

Section and Topic	Item #	Checklist item	Location where item is reported
	13e	Describe any methods used to explore possible causes of heterogeneity among study results (e.g. subgroup analysis, meta-regression).	Section 2.6
	13f	Describe any sensitivity analyses conducted to assess robustness of the synthesized results.	Section 2.6
Reporting bias assessment	14	Describe any methods used to assess risk of bias due to missing results in a synthesis (arising from reporting biases).	Section 2.6
Certainty assessment	15	Describe any methods used to assess certainty (or confidence) in the body of evidence for an outcome.	Section 2.7
**RESULTS**			
Study selection	16a	Describe the results of the search and selection process, from the number of records identified in the search to the number of studies included in the review, ideally using a flow diagram.	Section 3.1; Figure 1
	16b	Cite studies that might appear to meet the inclusion criteria, but which were excluded, and explain why they were excluded.	Supplementary Table S2, pp. 7–10
Study characteristics	17	Cite each included study and present its characteristics.	Section 3.2; Tables 1 and 2
Risk of bias in studies	18	Present assessments of risk of bias for each included study.	Section 3.3; Supplementary Figure S1
Results of individual studies	19	For all outcomes, present, for each study: (a) summary statistics for each group (where appropriate) and (b) an effect estimate and its precision (e.g. confidence/credible interval), ideally using structured tables or plots.	Figures 2–5; Supplementary Figures S2–S5
Results of syntheses	20a	For each synthesis, briefly summarise the characteristics and risk of bias among contributing studies.	Sections 3.4–3.7
	20b	Present results of all statistical syntheses conducted. If meta-analysis was done, present for each the summary estimate and its precision (e.g. confidence/credible interval) and measures of statistical heterogeneity. If comparing groups, describe the direction of the effect.	Table 3; Supplementary Table S3
	20c	Present results of all investigations of possible causes of heterogeneity among study results.	Supplementary Tables S5–S7
	20d	Present results of all sensitivity analyses conducted to assess the robustness of the synthesized results.	Section 3.8; Supplementary Tables S4, S8; Supplementary Figure S6
Reporting biases	21	Present assessments of risk of bias due to missing results (arising from reporting biases) for each synthesis assessed.	Section 3.9; Supplementary Figure S7
Certainty of evidence	22	Present assessments of certainty (or confidence) in the body of evidence for each outcome assessed.	Supplementary Table S1; Table 3
**DISCUSSION**			
Discussion	23a	Provide a general interpretation of the results in the context of other evidence.	Sections 4.1–4.6
	23b	Discuss any limitations of the evidence included in the review.	Section 4.7
	23c	Discuss any limitations of the review processes used.	Section 4.7
	23d	Discuss implications of the results for practice, policy, and future research.	Section 5
**OTHER INFORMATION**			
Registration and	24a	Provide registration information for the review, including register name and registration number, or state that	Section 2.1 (PROSPERO CRD420261371334)

PRISMA 2020 Checklist

Section and Topic	Item #	Checklist item	Location where item is reported
protocol		the review was not registered.	
	24b	Indicate where the review protocol can be accessed, or state that a protocol was not prepared.	Section 2.1 (PROSPERO CRD420261371334)
	24c	Describe and explain any amendments to information provided at registration or in the protocol.	Methods, Section 2.1
Support	25	Describe sources of financial or non-financial support for the review, and the role of the funders or sponsors in the review.	Funding section
Competing interests	26	Declare any competing interests of review authors.	Conflicts of Interest section
Availability of data, code and other materials	27	Report which of the following are publicly available and where they can be found: template data collection forms; data extracted from included studies; data used for all analyses; analytic code; any other materials used in the review.	Data Availability Statement; Supplementary Material (search strategies); PROSPERO protocol publicly accessible

From: Page MJ, McKenzie JE, Bossuyt PM, Boutron I, Hoffmann TC, Mulrow CD, et al. The PRISMA 2020 statement: an updated guideline for reporting systematic reviews. BMJ 2021;372:n71. doi: 10.1136/bmj.n71. This work is licensed under CC BY 4.0. To view a copy of this license, visit https://creativecommons.org/licenses/by/4.0/
